# Wells–Dawson phosphotungstates as mushroom tyrosinase inhibitors: a speciation study

**DOI:** 10.1038/s41598-021-96491-5

**Published:** 2021-09-29

**Authors:** Raphael Lampl, Joscha Breibeck, Nadiia I. Gumerova, Mathea Sophia Galanski, Annette Rompel

**Affiliations:** 1 Fakultät für Chemie, Institut für Biophysikalische Chemie, Universität Wien, Althanstraße 14, 1090 Wien, Austria; 2 Fakultät für Chemie, Institut für Anorganische Chemie und NMR Zentrum, Universität Wien, Währinger Str. 42, 1090 Wien, Austria

**Keywords:** Biochemistry, Bioinorganic chemistry, Metalloproteins, Inorganic chemistry

## Abstract

In order to elucidate the active polyoxotungstate (POT) species that inhibit fungal polyphenol oxidase (*Ab*PPO4) in sodium citrate buffer at pH 6.8, four Wells–Dawson phosphotungstates [*α*/*β*-P^V^_2_W^VI^_18_O_62_]^6−^ (intact form), [*α*_*2*_-P^V^_2_W^VI^_17_O_61_]^10−^ (monolacunary), [P^V^_2_W^VI^_15_O_56_]^12−^ (trilacunary) and [H_2_P^V^_2_W^VI^_12_O_48_]^12−^ (hexalacunary) were investigated. The speciation of the POT solutions under the dopachrome assay (50 mM Na-citrate buffer, pH 6.8; *L*-3,4−dihydroxyphenylalanine as a substrate) conditions were determined by ^183^W-NMR, ^31^P-NMR spectroscopy and mass spectrometry. The intact Wells–Dawson POT [*α*/*β*-P^V^_2_W^VI^_18_O_62_]^6−^ shows partial (~ 69%) disintegration into the monolacunary [*α*_*2*_-P^V^_2_W^VI^_17_O_61_]^10−^ anion with moderate activity (*K*_*i*_ = 9.7 mM). The monolacunary [*α*_*2*_-P^V^_2_W^VI^_17_O_61_]^10−^ retains its structural integrity and exhibits the strongest inhibition of *Ab*PPO4 (*K*_*i*_ = 6.5 mM). The trilacunary POT [P^V^_2_W^VI^_15_O_56_]^12−^ rearranges to the more stable monolacunary [*α*_*2*_-P^V^_2_W^VI^_17_O_61_]^10−^ (~ 62%) accompanied by release of free phosphates and shows the weakest inhibition (*K*_*i*_ = 13.6 mM). The hexalacunary anion [H_2_P^V^_2_W^VI^_12_O_48_]^12−^ undergoes time-dependent hydrolysis resulting in a mixture of [H_2_P^V^_2_W^VI^_12_O_48_]^12−^, [P^V^_8_W^VI^_48_O_184_]^40−^, [P^V^_2_W^VI^_19_O_69_(H_2_O)]^14−^ and [*α*_*2*_-P^V^_2_W^VI^_17_O_61_]^10−^ which together leads to comparable inhibitory activity (*K*_*i*_ = 7.5 mM) after 48 h. For the solutions of [*α*/*β*-P^V^_2_W^VI^_18_O_62_]^6−^, [*α*_*2*_-P^V^_2_W^VI^_17_O_61_]^10−^ and [P^V^_2_W^VI^_15_O_56_]^12−^ the inhibitory activity is correlated to the degree of their rearrangement to [*α*_*2*_-P^V^_2_W^VI^_17_O_61_]^10−^. The rearrangement of hexalacunary [H_2_P^V^_2_W^VI^_12_O_48_]^12−^ into at least four POTs with a negligible amount of monolacunary anion interferes with the correlation of activity to the degree of their rearrangement to [*α*_*2*_-P^V^_2_W^VI^_17_O_61_]^10−^. The good inhibitory effect of the Wells–Dawson [*α*_*2*_-P^V^_2_W^VI^_17_O_61_]^10−^ anion is explained by the low charge density of its protonated forms H_*x*_[*α*_*2*_-P^V^_2_W^VI^_17_O_61_]^(10−*x*)−^ (*x* = 3 or 4) at pH 6.8.

## Introduction

Polyphenol oxidases (PPOs) are copper-containing proteins omnipresent in animals, fungi, plants and bacteria^1–5^, with tyrosinases and catechol oxidases being prominent members of this enzyme family. Tyrosinases exhibit cresolase activity (EC 1.14.18.1; *ortho*-hydroxylation of monophenols to *ortho*-diphenols, monophenolase activity) and catecholase activity (EC 1.10.3.1; oxidation of *ortho*-diphenols to *ortho*-quinones; diphenolase activity), give rise to the rate determining step in melanogenesis and are involved in pigment coating and browning^6^. For its negligible lag phase and generally higher reaction velocity, the diphenolase activity of tyrosinases is usually assayed using *L*-DOPA (*L*-3,4−dihydroxyphenylalanine, Figure S1A), as the substrate for the dopachrome assay^7^. The polyoxotungstate (POT) inhibition parameters were determined by fitting the kinetic data to a generalized Michaelis–Menten^8^ model and Lineweaver–Burk plots^9^, accompanied by POT speciation studies applying ^31^P-NMR and ^183^W-NMR spectroscopy and mass spectrometry under physiological conditions (50 mM Na-citrate buffer, pH 6.8) with or without the substrate *L*-DOPA. A good mushroom PPO inhibitor is the structurally related kojic acid (Fig. S1B), which holds inhibition constant (*K*_*i*_)-values in the µM range^10^ and acts as a competitive inhibitor.^10^ Mushroom (*Agaricus bisporus*; abbr: *Ab*) PPO is present in great quantity in fruiting bodies^11^. The enzyme has been thoroughly characterized in its structure^12,13^ and activity^14^. Following the established protocol by Pretzler et al.^15^, *Ab*PPO4 was recombinantly expressed in *E. coli* and purified in its active form for the here presented inhibition studies.

Polyoxometalates (POMs) are metal–oxygen clusters commonly built up by W, Mo or V addenda ions, which are usually in their highest oxidation states exhibiting the electronic configuration d^0^ or d^1^^16,17^. POMs show a widespread biological applicability such as antibacterial^18^ and anti-tumor activity^19^, and stable POTs have successfully been applied as additives in co-crystallization experiments with proteins^20–24^. Apart from the Anderson-Evans archetype^25^ (e.g. [Te^VI^W^VI^_6_O_24_]^6−^ is stable in aqueous solution between pH 4.5 and 7.5) most POTs show low stability at physiological conditions, requiring solution NMR-measurements to reveal the composition under the applied conditions. As POTs possess a higher chemical stability than polyoxomolybdates (POMos), tungsten is usually selected as the addenda ion, when applying this compound class in biological investigations^26^.

Recently, a systematic approach varying the charge density in a series of Keggin POTs was reported by Breibeck et al.^27^ to characterize their inhibitory effects against recombinant *Ab*PPO4^15^. A detailed assignment of the active Keggin POT species was undertaken applying NMR spectroscopy^27^. A charge density dependence of the inhibitory capacities was derived, where the most active Keggin POT was [Si^IV^W^VI^_12_O_40_]^4−^ (*K*_*i*_ = 4.7 mM), and the more stable Keggin anions with higher charge density did not inhibit diphenolase activity.

**Figure 1:**
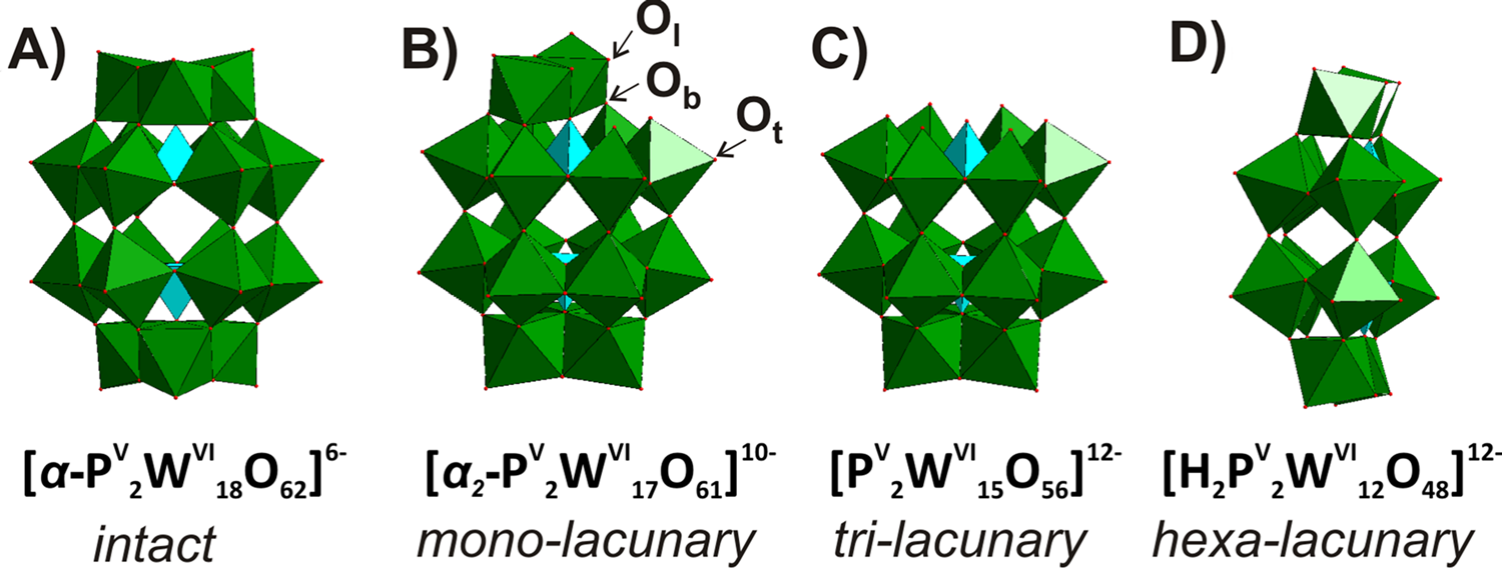
The POTs (**A**–**D**) feature the Wells–Dawson archetype and they are derived from the *α*-isomer of [P^V^_2_W^VI^_18_O_62_]^6−^. Three types of oxygen atoms (O_t_—terminal, O_b_—bridging, O_l_—surrounding the lacuna) are exemplified in (**B**). Color code*:* {WO_6_} octahedra, green; {PO_4_} tetrahedra, turquoise.

Another prominent POM scaffold investigated for biological activity is the Wells–Dawson archetype with the general formula [(X^*n*+^)_2_M_18_O_62_]^(16−2*n*)−^^28^, where M is either W^VI^ or Mo^VI^ and X^*n*+^ is commonly phosphorus(V) or arsenic(V). For the intact form of this cluster, 18 {MO_6_} octahedral units enclose both inner heteroion groups {XO_4_} by corner- and edge-sharing (Fig. 1). Wells–Dawson POMs exhibited antibacterial^29^ activity, and both the intact anion [*α*/*β*-P^V^_2_W^VI^_18_O_62_]^6−^ and the hexalacunary form [H_2_P^V^_2_W^VI^_12_O_48_]^12−^ inhibited Ca^2+^-ATPase, P-type ATPase^30^, and aquaporin-3^31^. Transition metal-substituted Wells–Dawson POMos were used as inhibitors against α-glucosidase^32^ with *K*_*i*_-values covering a wide range down to the µM scale.

In this study, the inhibitory effect of four structurally related Wells–Dawson POTs, intact Cat_6_[*α*/*β*-P^V^_2_W^VI^_18_O_62_]·14H_2_O (Cat = K^+^, NH_4_^+^)^33,34^, mono-lacunary K_10_[*α*_2_-P^V^_2_W^VI^_17_O_61_]·20H_2_O^34,35^, tri-lacunary K_12_[P^V^_2_W^VI^_15_O_56_]·24H_2_O^35,36^ and hexalacunary (NH_4_)_12_[H_2_P^V^_2_W^VI^_12_O_48_]·24H_2_O^37,38^, with net charges from 6− to 12− (Fig. 1) on the catecholase activity of *Ab*PPO4 is explored. The focus is on the identification of the active species present under the diphenolase activity assay conditions by NMR spectroscopy and mass spectrometry. For the inhibition studies, the POTs were buffered at pH 6.8 in 50 mM Na-citrate and the obtained solutions are termed [**P**_**2**_**W**_**18**_]^6−^ for K_6_[*α*/*β*-P^V^_2_W^VI^_18_O_62_]·14H_2_O and (NH_4_)_6_[*α*/*β*-P^V^_2_W^VI^_18_O_62_]·14H_2_O, [**P**_**2**_**W**_**17**_]^10−^ for K_10_[*α*_2_-P^V^_2_W^VI^_17_O_61_]·20H_2_O, [**P**_**2**_**W**_**15**_]^12−^ for K_12_[P^V^_2_W^VI^_15_O_56_]·24H_2_O and [**P**_**2**_**W**_**12**_]^12−^ for (NH_4_)_12_[H_2_P^V^_2_W^VI^_12_O_48_]·24H_2_O to distinguish the buffered solution sample from the solid compound (see Abbreviation section in SI).

## Results and discussion

### Activity plots of *Ab*PPO4 inhibited by Wells–Dawson POTs

**Figure 2:**
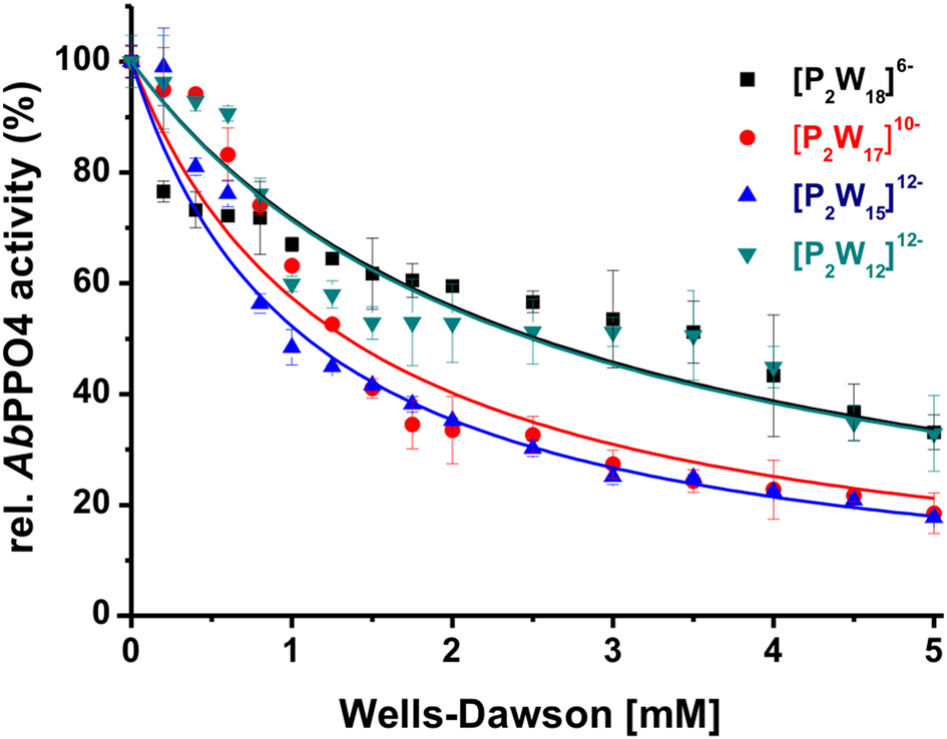
Activity plots of *Ab*PPO4 with four Wells–Dawson phosphotungstates [**P**_**2**_**W**_**18**_]^6−^, [**P**_**2**_**W**_**17**_]^10−^, [**P**_**2**_**W**_**15**_]^12−^ and [**P**_**2**_**W**_**12**_]^12−^. The dopachrome assay was performed using 1 mM *L*-DOPA as the substrate in 50 mM Na-citrate buffer at pH 6.8. The measurements were taken in triplicates and a hyperbolic curve fit (Eq. (5), SI) was performed. The kinetic inhibition parameters are summarized in Table 1.

*Ab*PPO4 was purified and activated according to Pretzler et al.^15^. The ESI–MS of active *Ab*PPO4 is presented in Fig. S2 and Table S1, the protein sequence of *Ab*PPO4 in Fig. S3 and the SDS-PAGE analysis of *Ab*PPO4 is shown in Figs. S4 and S5. The protocols for the synthesis of the Wells–Dawson POTs were taken from published procedures (cf. Table S2) and their identity was confirmed by IR (Fig. S6, Table S3) in the solid state and by ^31^P- and ^183^W-NMR in solutions (Figs. S7–S13). To investigate the Wells–Dawson POT-mediated inhibition of *Ab*PPO4, the corresponding POT ([**P**_**2**_**W**_**18**_]^6−^, [**P**_**2**_**W**_**17**_]^10−^, [**P**_**2**_**W**_**15**_]^12−^, [**P**_**2**_**W**_**12**_]^12−^) was dissolved in 50 mM Na-citrate buffer (pH 6.8), where the enzyme shows its maximal activity^4,15^. The diphenolase activity of tyrosinase was monitored using 1 mM *L*-DOPA (Fig. S1A) as the substrate. Considering that the Wells–Dawson POT stability is pH and possibly time dependent, a speciation study was performed which is detailed in the paragraph "Speciation of Wells–Dawson POT by ^183^W-NMR and ^31^P-NMR analyses at pH 6.8". The concentration of the investigated Wells–Dawson phosphotungstates was in the range between 0 and 5 mM. If enzymatic inhibition was observed, data were taken in triplicates and fitted with a hyperbolic function (cf. SI Eq. (5)) from a mixed inhibition model to evaluate the *K*_*i*_ and α-parameters. All four solutions [**P**_**2**_**W**_**18**_]^6−^, [**P**_**2**_**W**_**17**_]^10−^, [**P**_**2**_**W**_**15**_]^12−^ and [**P**_**2**_**W**_**12**_]^12−^ showed a mixed-type inhibition with *K*_*i*_ values in the mM range, binding both to the free enzyme and to the enzyme–substrate complex^39^ (Table 1, Fig. 2). As a positive control for the inhibition of *Ab*PPO4 diphenolase activity and for validation of the kinetic methodology, the well-characterized natural PPO-inhibitor kojic acid^10^ (Fig. S1B) was additionally tested and evaluated applying exactly the same mathematical model. The organic inhibitor kojic acid features a *K*_*i*_ in the µM range corresponding to a much higher affinity to the enzyme as revealed for the Wells–Dawson POTs. The *K*_*i*_ values previously reported for Keggin POTs [XW^VI^_12_O_40_]^n−^ (X = P^V^, Si^IV^, B^III^, A^lII^, H_2_^2+^, Be^II^), which were obtained under the same conditions, are also in the mM range and vary from 4.7 mM (X = Si^IV^) to 25.6 mM (X = P^V^)^27^. The *K*_*i*_ values obtained using the Michaelis–Menten model, which is the generally accepted method for calculating the enzyme kinetic parameters, are used as primary values to compare the POTs’ activities.

**Table 1:** Summary of kinetic evaluation of *Ab*PPO4 inhibition by Wells–Dawson POTs.

Inhibitor	*K*_*i*_ [mM]	*α*	R^2^	Inhibition type
[**P**_**2**_**W**_**18**_]^6−^	9.7^a^, 13.0^b^	0.013^a^, 0.22^b^	0.80	Mixed-type
[**P**_**2**_**W**_**17**_]^10−^	6.5^a^, 10.6^b^	0.01^a^, 0.01^b^	0.93	Mixed-type
[**P**_**2**_**W**_**15**_]^12−^	13.6^a^, 10.7^b^	0.003^a^, 0.20^b^	0.96	Mixed-type
[**P**_**2**_**W**_**12**_]^12−^	7.5^a^, 5.6^b^	0.017^a^, 0.28^b^	0.91	Mixed-type
Control: kojic acid ^10^	4.5·10^–3a^, 4.3·10^–3b^	2.6 × 10^15^	1.0	Competitive

*K*_*i*_: inhibition constant. *α*: inhibition parameter, R^2^: curve fit determination coefficient; the parameters were determined ^a^from activity plot and ^b^from the Lineweaver–Burk slopes or intercepts. For comparison, the results for the Keggin series are provided in^27^.

To evaluate the inhibitory effect, the activity curves for four Wells–Dawson phosphotungstates were plotted in the concentration range 0–5 mM (Fig. 2). Among the Wells–Dawson clusters, [**P**_**2**_**W**_**17**_]^10−^ showed the greatest inhibitory effect (*K*_*i*_ = 6.5 mM) and the fitted *α*-parameter suggests a mixed mode of inhibition (Table 1). According to their *K*_*i*_ values [**P**_**2**_**W**_**17**_]^10−^ (*K*_*i*_ = 6.5 mM) and [**P**_**2**_**W**_**12**_]^12−^ (*K*_*i*_ = 7.5 mM) exhibited nearly identical inhibitory activity and [**P**_**2**_**W**_**18**_]^6−^ (*K*_*i*_ = 9.7 mM) and [**P**_**2**_**W**_**15**_]^12−^ (*K*_*i*_ = 13.6 mM) showed a lower inhibition capacity.

### Lineweaver–Burk evaluation of inhibition types

The type of enzymatic inhibition is usually investigated by linear plots according to Lineweaver–Burk, also allowing for further validation of the inhibitory constant *K*_*i*_.^9^ For [**P**_**2**_**W**_**18**_]^6−^, [**P**_**2**_**W**_**17**_]^10−^, [**P**_**2**_**W**_**15**_]^12−^ and [**P**_**2**_**W**_**12**_]^12−^ as well as for the kojic acid control measured in our previous study^27^, the dopachrome assay was repeated at five different substrate concentrations (varying from 0.4 to 1.5 mM) and three different inhibitor concentrations, respectively (SI section 7, Figs. S14–S17, Tables S4, S5). Each POT analysis yielded a set of three lines intersecting in a common point. The slopes (SI Eq. (13), insets in Figs. S14–S17) and ordinate intercepts of these regression lines were further evaluated to validate the inhibitory constant *K*_*i*_ and the α-parameter from the non-linear regression procedure. Therefore, the slopes were plotted against the used inhibitor concentrations to give lines intersecting the abscissa at -*K*_*i*_. Similarly, the Lineweaver–Burk ordinate intercepts were evaluated for the α-parameter (cf. Table S5 and Table 1). In good accordance with their structural similarity, the respective intersection point of the three Lineweaver–Burk lines is the third quadrant for all four Wells–Dawson POT solutions [**P**_**2**_**W**_**18**_]^6−^, [**P**_**2**_**W**_**17**_]^10−^, [**P**_**2**_**W**_**15**_]^12−^ and [**P**_**2**_**W**_**12**_]^12−^, which indicated mixed-type inhibition^39,40^ previously found for POT representatives of the Keggin archetype^27^.

### Speciation of Wells–Dawson POT by ^183^W-NMR and ^31^P-NMR analyses at pH 6.8

**Figure 3:**
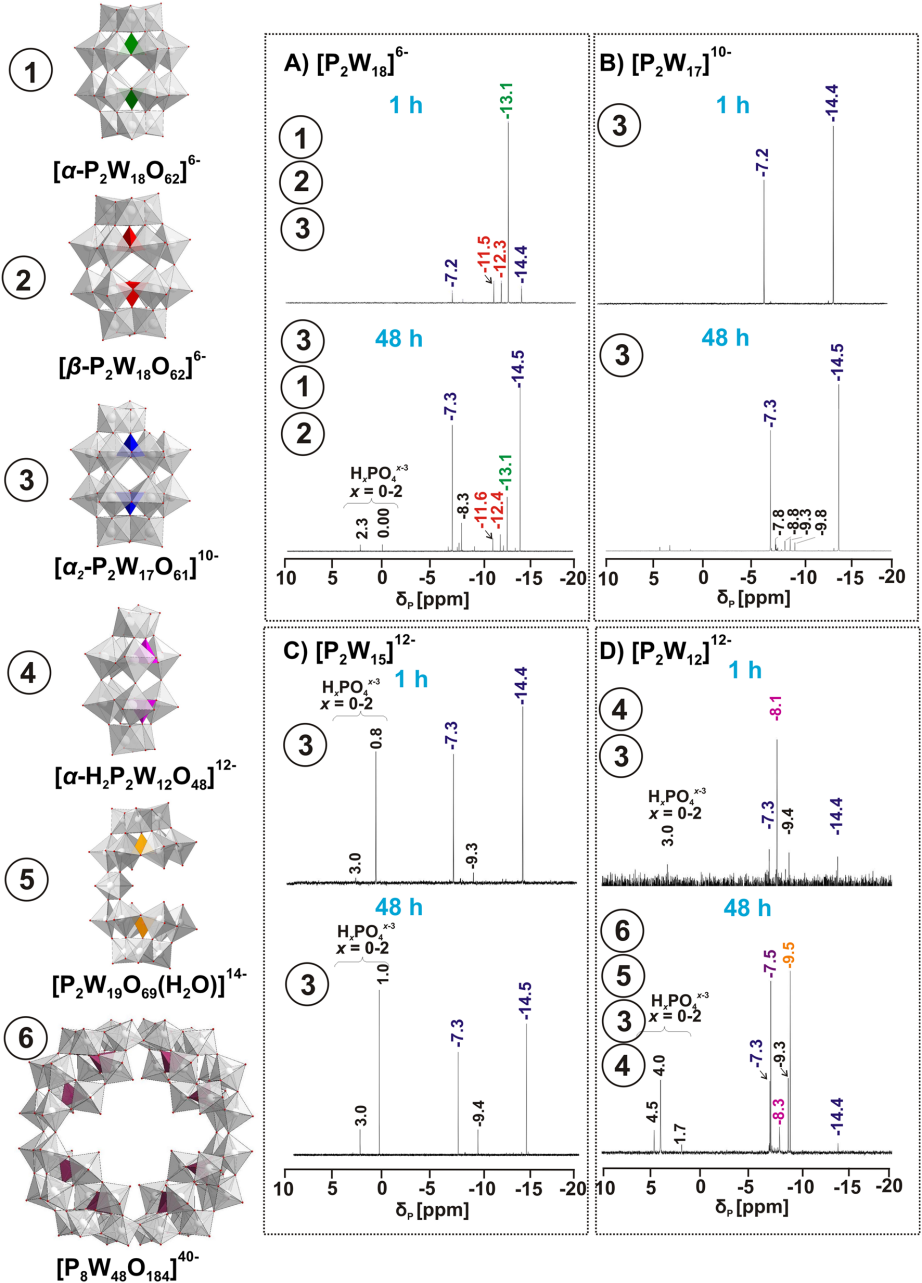
^31^P-NMR spectra of [**P**_**2**_**W**_**18**_]^**6−**^, [**P**_**2**_**W**_**17**_]^**10−**^, [**P**_**2**_**W**_**15**_]^**12−**^ and [**P**_**2**_**W**_**12**_]^**12−**^ in 50 mM Na-citrate buffer at pH 6.8 (Table S2) recorded 1 h and 48 h after preparation. A) educts: [*α*-P^V^_2_W^VI^_18_O_62_]^6−^ and [*β*-P^V^_2_W^VI^_18_O_62_]^6−^; products: *fresh*—[*α/β* -P^V^_2_W^VI^_18_O_62_]^6−^ (88%) and [*α*_*2*_-P^V^_2_W^VI^_17_O_61_]^10−^ (12%), *after 48 h* – [*α/β* -P^V^_2_W^VI^_18_O_62_]^6−^ (15%) and [*α*_*2*_-P^V^_2_W^VI^_17_O_61_]^10−^ (69%); B) educt [*α*_*2*_-P^V^_2_W^VI^_17_O_61_]^10−^; products: *fresh*—[*α*_*2*_-P^V^_2_W^VI^_17_O_61_]^10−^ (100%), *after 48 h*—[*α*_*2*_-P^V^_2_W^VI^_17_O_61_]^10−^ (92%); C) educt: [P^V^_2_W^VI^_15_O_56_]^12−^; products: *fresh*—[*α*_*2*_-P^V^_2_W^VI^_17_O_61_]^10−^ (62%), *after 48 h*—[*α*_*2*_-P^V^_2_W^VI^_17_O_61_]^10−^ (61%); D) educt: [H_2_P^V^_2_W^VI^_12_O_48_]^12−^; products: *fresh*—[H_2_P^V^_2_W^VI^_12_O_48_]^12−^ (72%), [*α*_*2*_-P^V^_2_W^VI^_17_O_61_]^10−^ (20%), *after 48 h*—[P^V^_8_W^VI^_48_O_184_]^40−^ (23%), [P^V^_2_W^VI^_19_O_69_(H_2_O)]^14−^ (22%), [H_2_P^V^_2_W^VI^_12_O_48_]^12−^ (9%), [*α*_*2*_-P^V^_2_W^VI^_17_O_61_]^10−^ (3%). Weak signals in the range between − 10 and − 7 ppm correspond to unstable solution intermediates with a total of 9% for [**P**_**2**_**W**_**18**_]^6−^ (**A**); 8% for [**P**_**2**_**W**_**17**_]^10−^ (**B**); 5% for [**P**_**2**_**W**_**15**_]^12−^ (**C**) and 19% for [**P**_**2**_**W**_**12**_]^12−^ (**D**) in aged solutions based on integrated POT signals^34,41,44^, Signal assignment is based on references^34,37,41^. Color code*:* {WO_6_} octahedra, white; {PO_4_} tetrahedra, green, red, blue, pink, orange, burgundy; O atoms, red.

The detailed structural information about species under the respective experimental conditions is essential for understanding of POT activity^17^. All Wells–Dawson POTs were dissolved in reaction buffer (50 mM Na-citrate, pH 6.8) and were adjusted with 1 M HCl or 1 M NaOH to pH 6.8 for measurements with *Ab*PPO4, where the enzyme shows its maximal activity^4,15^. Upon dissolution of [**P**_**2**_**W**_**18**_]^6−^ and [**P**_**2**_**W**_**17**_]^10−^, the pH of the buffer slightly decreased, whereas [**P**_**2**_**W**_**15**_]^12−^ and [**P**_**2**_**W**_**12**_]^12−^ behaved as bases. ^31^P-NMR in combination with ^183^W-NMR is established as a useful technique to determine the structural composition of the Wells–Dawson POTs^16^ and was previously successfully applied to elucidate the speciation of Keggin—type POTs in an analogous study^27^. The concentration of all species was calculated based on ^31^P-NMR peaks integration. Since the stock solutions of each Wells–Dawson POT was used up to three days after preparation and ^183^W-NMR acquisition takes 60 h, ^31^P-NMR spectra for all four solutions have been recorded 1 h and 48 h after preparation to check if speciation changes occur over time. Under the experimental conditions (50 mM Na-citrate, pH 6.8) [**P**_**2**_**W**_**18**_]^6−^ showed partial hydrolysis of intact [*α*/*β*-P^V^_2_W^VI^_18_O_62_]^6−^ (Fig. 1A) to monolacunary (Fig. 1B) [*α*_*2*_-P^V^_2_W^VI^_17_O_61_]^10−^ (Figs. 3A, S8B). In the solution of intact POT one hour after preparation 12% of [*α*_*2*_-P^V^_2_W^VI^_17_O_61_]^10−^ was detected based on ^31^P-NMR peaks integration (Fig. 3A), whereas 48 h after dissolution the concentration of monolacunary anion increased to 69%. The ^183^W-NMR spectrum of [**P**_**2**_**W**_**18**_]^6−^ (Fig. S8B) is in agreement with ^31^P-NMR data and demonstrates 9 signals for the mono-lacunary anion and 6 additional signals related to a mixture of intact [*α*/*β*-P^V^_2_W^VI^_18_O_62_]^6−^ isomers. ^31^P-NMR studies showed that the solution of monolacunary POT [**P**_**2**_**W**_**17**_]^10−^ exhibited [*α*_*2*_-P^V^_2_W^VI^_17_O_61_]^10−^ as the only species present in the freshly prepared solution (Fig. 3B), and as the dominant anion (92%) after 48 h of solution aging. The ^31^P-NMR spectrum of tri-lacunary POT [**P**_**2**_**W**_**15**_]^12−^ (Fig. 3C) demonstrates the fast rearrangement of the tri-lacunary anion to 62% of monolacunary [*α*_*2*_-P^V^_2_W^VI^_17_O_61_]^10−^ in both fresh and in 48 h aged solutions with 35% of free phosphate anions H_*x*_PO_4_^*x*−3^ and 3% remaining unidentified phosphotungstates. The ^183^ W-NMR spectra of monolacunary [**P**_**2**_**W**_**17**_]^10−^ (Fig. S8A) and trilacunary [**P**_**2**_**W**_**15**_]^12−^ (Fig. S8C) are in agreement with ^31^P-NMR data confirming the presence of only the monolacunary anion by 9 signals. The ^31^P-NMR spectrum of fresh hexalacunary POT [**P**_**2**_**W**_**12**_]^12−^ (Fig. 3D) points to the presence of unhydrolyzed hexalacunary [H_2_P^V^_2_W^VI^_12_O_48_]^12−^ (72%) together with a low amount of monolacunary [*α*_*2*_-P^V^_2_W^VI^_17_O_61_]^10−^ (20%). After 48 h, the signals corresponding to hexalacunary [H_2_P^V^_2_W^VI^_12_O_48_]^12−^ and monolacunary [*α*_*2*_-P^V^_2_W^VI^_17_O_61_]^10−^ decreased to 9% and 3%, respectively, while two intense signals at − 7.5 and − 9.5 ppm appeared. These signals are of comparable intensities, but do not correspond to the monolacunary [*α*_*1*_-P^V^_2_W^VI^_17_O_61_]^10−^ isomer.^34^ An anion which can give rise to a signal at − 9.5 ppm is [P^V^_2_W^VI^_19_O_69_(H_2_O)]^14−^, which contains two *A*-*α*-[P^V^W^VI^_9_O_34_]^9-^ halves linked via one W^VI^ ion in the equatorial plane and was previously reported as an intermediate species in phosphotungstate solutions as well as isolated in the solid state^41,42^. The signal at − 7.5 ppm corresponds to [P^V^_8_W^VI^_48_O_184_]^40−^, which was previously detected in solutions with a high amount of NH _4_^+^^37^. The presence of four different POTs in [**P**_**2**_**W**_**12**_]^12−^ 48 h after preparation renders the detection of all species’ signals in the ^183^W-NMR spectrum impossible due to their low concentrations and the low abundancy (14%) of the ^183^W isotope (Fig. S8D). The most intense signals at -190.3, -191.6 and -211.3 ppm correspond to three types of W ions in cyclic [P^V^_8_W^VI^_48_O_184_]^40−^^43^. ^31^P-NMR spectra recorded 96 h after preparation are identical to those recorded after 48 h for [**P**_**2**_**W**_**18**_]^6−^, [**P**_**2**_**W**_**17**_]^10−^ and [**P**_**2**_**W**_**15**_]^12−^. In the ^31^P-NMR spectrum of [**P**_**2**_**W**_**12**_]^12−^, the signal at − 8.3 ppm corresponding to hexalacunary [H_2_P^V^_2_W^VI^_12_O_48_]^12−^ disappeared completely after 96 h. In every ^31^P-NMR analysis, peaks for free phosphate were observed, indicating the release of H_*x*_PO_4_^*x*−3^ from the POT clusters as a consequence of partial decomposition or rearrangement (Fig. 3). The weak signals between − 12 and − 7 ppm in the ^31^P-NMR spectra (Fig. 3) with a total amount of no more than 19% of all POT signals, correspond to solution intermediates of unknown identity that have been reported previously^44^. The inhibitory activity of Wells–Dawson solutions does not correlate with the presence of any of those intermediates.

### Influence of the substrate *L*-DOPA at pH 6.8 on POT's speciation

During the kinetic measurement of intact [**P**_**2**_**W**_**18**_]^6−^, the solution immediately turned blue due to the intervalence charge transfer of W^V^–O–W^VI^ ↔ W^VI^–O–W^V^ after addition of *L*-DOPA, hinting towards the reduction of POTs in the presence of *L*-DOPA (Fig. S18). Many POTs are redox active and can easily accept electrons^45^. The cyclic voltammogram of the intact Wells–Dawson anion [*α*-P^V^_2_W^VI^_18_O_62_]^6−^ displays notable proton-coupled electron redox activity, which allows this molecule to reversibly accept up to 18 electrons in aqueous solution at pH 4^46^. In contrast, lacunary anions (Fig. 1B–D) accept electrons less easily, for example, the reduction energy of monolacunary [*α*_2_-P_2_W^VI^_17_O_61_]^10−^ is less favorable by 1.6 eV than for intact [P^V^_2_W^VI^_18_O_62_]^6−^^47^. Since a reduction occurs only in solution of intact [**P**_**2**_**W**_**18**_]^6−^ in the presence of *L*-DOPA and no color change and no change in speciation (Figs. S9, S11, Table S2) were observed for the other three POT samples [**P**_**2**_**W**_**17**_]^10−^, [**P**_**2**_**W**_**15**_]^12−^ and [**P**_**2**_**W**_**12**_]^12−^, it can be concluded that only the intact [P^V^_2_W^VI^_18_O_62_]^6−^ accepts electrons in the presence of *L*-DOPA. This reduction and protonation from [P^V^_2_W^VI^_18_O_62_]^6−^ to H_2_[P^V^_2_W^VI^_12_W^V^_6_O_62_]^10−^ has been previously reported at pH 6.8^48^. The reduction might affect inhibitory activity of [**P**_**2**_**W**_**18**_]^6−^ based on the lower value of R^2^ for [**P**_**2**_**W**_**18**_]^6−^ as compared to the ones observed for the other Wells–Dawson POTs [**P**_**2**_**W**_**17**_]^10−^, [**P**_**2**_**W**_**15**_]^12−^ and [**P**_**2**_**W**_**12**_]^12−^ (Table 1).

### Charge density dependence of inhibitory effects of Wells–Dawson POT species

The charge density of POMs, expressed in number of charges (*q*) per addenda metal ions (*m*), is a criterion to characterize the chaotropic behavior of POMs (Table S6)^49–53^. Recently, it was shown that the affinity of POMs towards biomolecules is attributable to their superchaotropic character, and POMs with moderate charge densities (*q/m* = 0.33) interact considerably strong with surfaces of different or mixed polarities, which are present in protein molecules^54^. The activity of [**P**_**2**_**W**_**18**_]^6−^, [**P**_**2**_**W**_**17**_]^10−^ and [**P**_**2**_**W**_**15**_]^12−^ solutions correlates with the amount of the dominant monolacunary anion [*α*_*2*_-P^V^_2_W^VI^_17_O_61_]^10−^ in Na-citrate buffer at pH 6.8 (Fig. 3, Scheme S1). For the unprotonated form of the active monolacunary Wells–Dawson anion [*α*_*2*_-P^V^_2_W^VI^_17_O_61_]^10−^ the *q/m* ratio is 0.59 (Table 2), however, the protonation of this anion in neutral solutions has previously been shown by electrochemical analysis in combination with theoretical calculations^47,55^. For POMs the proton affinity difference between the terminal and bridging oxygen is around 11 kcal mol^−1^^56^, proving that the four oxygen atoms surrounding the lacuna are much more basic than the bridging and terminal O atoms (Fig. 1B), and thus prone to protonation. Electrospray-ionization mass spectrometry (ESI–MS) has already been successfully used for the protonation states assignment of Wells–Dawson POTs containing different heteroatoms^57^. Consequently, ESI–MS has been applied to investigate the protonation state of monolacunary [*α*_*2*_-P^V^_2_W^VI^_17_O_61_]^10−^. When recording ESI–MS for POMs in Na-citrate buffer, the relative intensity of citrate anion signals is almost 100%, suppressing POM signals. Hence, the measurements had to be carried out in water at pH 6.4 (adjusted with HCl), where [*α*_*2*_*-*P^V^_2_W^VI^_17_O_61_]^10−^ is the predominant species (Fig. S19), as well as in a CH_3_CN/CH_3_OH/H_2_O mixture. The spectra recorded in both solvents show signals for NaH_4_[*α*_*2*_-P^V^_2_W^VI^_17_O_61_]^5-^ at 838.0 and NaKH_3_[*α*_*2*_-P^V^_2_W^VI^_17_O_61_]^5-^ at 845.6 m/z, while species with lower protonation states have not been detected (Fig. S19). Therefore, the monolacunary anion [*α*_*2*_*-*P^V^_2_W^VI^_17_O_61_]^10−^ is present as the three-fold protonated anion H_3_[*α*_*2*_-P^V^_2_W^VI^_17_O_61_]^7-^ with a charge density of 0.41 and the four-fold protonated anion H_4_[*α*_*2*_-P^V^_2_W^VI^_17_O_61_]^6−^ with a charge density of 0.35. The predominant species in [**P**_**2**_**W**_**12**_]^12−^ is the wheel-shaped [P^V^_8_W^VI^_48_O_184_]^40−^ (23%), which can be protonated to H_16_[P^V^_8_W^VI^_48_O_184_]^24−^^58^ *q/m* = 0.5. Due to the compositional complexity of the hexalacunary anion it is impossible to accurately correlate its activity with the POTs’ charge density.

**Table 2:** Stability under physiological conditions and charge densities (*q/m*) of [**P**_**2**_**W**_**18**_]^6−^, [**P**_**2**_**W**_**17**_]^10−^, [**P**_**2**_**W**_**15**_]^12−^ and [**P**_**2**_**W**_**12**_]^12−^, with H_*x*_[*α*_*2*_-P^V^_2_W^VI^_17_O_61_]^(10−*x*)-^ (x = 3 or 4).

Educt POT	Charge density *q/m* of POT educt	Stable in Na-citrate buffer at pH 6.8	Dominant POT species at pH 6.8 in Na-citrate buffer	Charge density *q/m* of POT product
[*α*/*β*-P^V^_2_W^VI^_18_O_62_]^6−^	0.33	No	H_*x*_[*α*_*2*_-P^V^_2_W^VI^_17_O_61_]^(10−*x*)−^	0.35 for *x* = 4 and 0.41 for *x* = 3
[*α*_*2*_-P^V^_2_W^VI^_17_O_61_]^10−^	0.59	Yes	H_*x*_[*α*_*2*_-P^V^_2_W^VI^_17_O_61_]^(10−*x*)−^	0.35 for *x* = 4 and 0.41 for *x* = 3
[P^V^_2_W^VI^_15_O_56_]^12−^	0.8	No	H_*x*_[*α*_*2*_-P^V^_2_W^VI^_17_O_61_]^(10−*x*)−^	0.35 for *x* = 4 and 0.41 for *x* = 3
H_2_[P^V^_2_W^VI^_12_O_48_]^12−^	1	No	[H_2_P^V^_2_W^VI^_12_O_48_]^12−^ + H_*x*_[*α*_*2*_-P^V^_2_W^VI^_17_O_61_]^(10−*x*)−^ + [P^V^_2_W^VI^_19_O_69_(H_2_O)]^14−^ + [P^V^_8_W^VI^_48_O_184_]^40−^	–

## Conclusions

**Figure 4:**
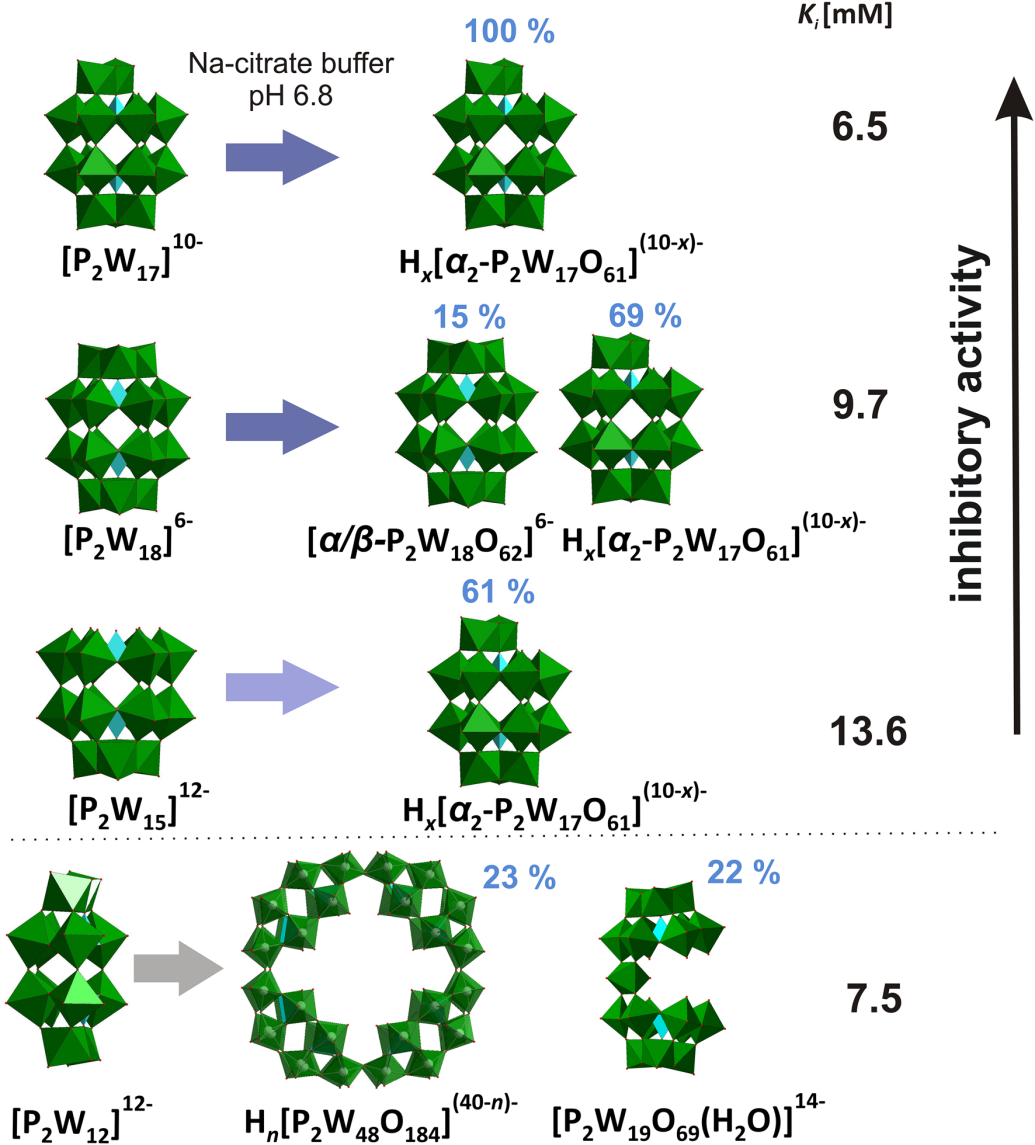
The POTs’ inhibitory activities are compared with the main species at pH 6.8 and at assay conditions. The intensity of the blue arrows correlates with the relative rearrangement to H_*x*_[*α*_*2*_-P^V^_2_W^VI^_17_O_61_]^(10−*x*)−^ (*x* = 3 or 4) for the different inhibitors at pH 6.8. The more H_*x*_[*α*_*2*_-P^V^_2_W^VI^_17_O_61_]^(10−*x*)−^ (*x* = 3 or 4) is in the sample, the higher the observed inhibitory activity of the POT. For [**P**_**2**_**W**_**12**_]^12−^ two predominant species are shown. *n* = 7–16 in H_*n*_[P^V^_8_W^VI^_48_O_184_]^(40−n)-^ according to^58^. Color code*:* {WO_6_} octahedra, green; {PO_4_} tetrahedra, turquoise.

The inhibitory effects of four Wells–Dawson phosphotungstates starting from intact, mono-, three- and hexalacunary forms against *Ab*PPO4 were investigated with a focus on speciation under the dopachrome assay conditions. During the investigation of [**P**_**2**_**W**_**18**_]^6−^, [**P**_**2**_**W**_**17**_]^10−^ and [**P**_**2**_**W**_**15**_]^12−^, the inhibition effect was assigned to the stable POT species H_*x*_[*α*_*2*_-P^V^_2_W^VI^_17_O_61_]^(10−*x*)−^ (*x* = 3 or 4), to which intact [*α*/*β*-P^V^_2_W^VI^_18_O_62_]^6−^ and trilacunary [P^V^_2_W^VI^_15_O_56_]^12−^ rearrange in significant quantities (Fig. 4). Interestingly, hexalacunary [**P**_**2**_**W**_**12**_]^12−^ demonstrates a more complex and time-dependent scenario with re-arrangement to the mixture of [P^V^_8_W^VI^_48_O_184_]^40−^ (23%), [P^V^_2_W^VI^_19_O_69_(H_2_O)]^14−^ (22%), [H_2_P^V^_2_W^VI^_12_O_48_]^12−^ (9%), [*α*_*2*_-P^V^_2_W^VI^_17_O_61_]^10−^ (3%) and free phosphate (24%), which does not allow us to unambiguously assign activity to one or several POT anions. The magnitude of the inhibitory activities correlated with the amount of H_*x*_[*α*_*2*_-P^V^_2_W^VI^_17_O_61_]^(10−*x*)-^ (*x* = 3 or 4) in the case of [**P**_**2**_**W**_**18**_]^6−^ (*K*_*i*_ = 9.7 mM), [**P**_**2**_**W**_**17**_]^10−^ (*K*_*i*_ = 6.5 mM) and [**P**_**2**_**W**_**15**_]^12−^ (*K*_*i*_ = 7.5 mM), which was quantified by ^31^P-NMR peaks integration.

## Methods

Protocols for synthesis, spectroscopic assignments and kinetic curves are discussed in the supplementary information. All chemicals have been purchased from Sigma-Aldrich (Vienna, Austria) and Carl-Roth (Karlsruhe, Germany) and were at least of analytical grade. They were used without further purification.

### Preparation of *Ab*PPO4 in its active form

For the preparation of active *Ab*PPO4, the procedure published by Pretzler et al.^15^ was used. Briefly, latent (inactive) *Ab*PPO4 was expressed with an N-terminal glutathione-S-transferase tag in *E. coli* BL21(DE3). For the protein expression, ZYM-5052 medium^59^ without addition of trace elements was used for 20 h at 20 °C, before 0.5 mM CuSO_4_ was added to the medium and incubation was continued for another 20 h. The cells were resuspended in Tris/HCl buffer.

The cell lysis was done via French press. After centrifugation, the supernatant was loaded onto an affinity chromatography GSTrap Fast Flow column and GST-*Ab*PPO4 was obtained after elution with glutathione. The GST tag was cut by digestion with HRV-3C protease. After another affinity chromatography step the latent *Ab*PPO4 was obtained in the flow-through. For activation of latent *Ab*PPO4 for inhibition studies the C-terminal cap was removed from the PPO with Proteinase K and the activated form was purified via size-exclusion chromatography in 50 mM Na-citrate buffer at pH 6.8.

### Synthesis of α-Wells Dawson POTs

The Wells–Dawson POTs were synthesized according to the procedures given in Table S2 in the Supplementary Information. The starting K_6_[*α*/*β*-P^V^_2_W^VI^_18_O_62_]·14 H_2_O and (NH_4_)_6_[*α*/*β*-P^V^_2_W^VI^_18_O_62_]·14 H_2_O were synthesized as mixtures of alpha and beta isomers precisely following the procedure from ref.^34^. K_6_[*α*/*β*-P^V^_2_W^VI^_18_O_62_]·14 H_2_O and (NH_4_)_6_[*α*/*β*-P^V^_2_W^VI^_18_O_62_]·14 H_2_O are both suitable for synthesis of lacunary derivatives^34^. Slight modifications during the preparations are given below:

1. (NH_4_)_6_[*α*/*β*-P^V^_2_W^VI^_18_O_68_]·14H_2_O: During the synthesis no bromine water was added.
2. (NH_4_)_12_[*α*-H_2_P^V^_2_W^VI^_12_O_48_]·24H_2_O: The precipitate was collected after reaction overnight.

### IR spectroscopy

A Bruker Vertex 70 IR Spectrometer equipped with a single-reflection diamond-ATR unit was used to verify the structure of the applied POTs. The distortion vibrations of W–O–W arise at 400 to 900 cm^−1^, the W=O stretching vibration occurs in the range of 930–960 cm^−1^ and the P=O vibrations appear between 960 and 1200 cm^−1^ (Fig. S6, Table S3).

### Electrospray-ionization mass spectrometry

Analysis was performed with an ESI–Qq–oaRTOF supplied by Bruker Daltonics Ltd. Bruker Daltonics Data Analysis software was used to analyze the results. The measurements were carried out in H_2_O and in a mixture of CH_3_CN/MeOH/H_2_O, collected in negative ion mode and with the spectrometer calibrated with the standard tune—mix to give an accuracy of better than 5 ppm in the region of m/z 100–1900.

### Nuclear magnetic resonance spectroscopy

^183^W-NMR and ^31^P-NMR were recorded with a Bruker FT-NMR spectrometer Avance Neo 500 MHz (Bruker, Rheinstetten, Germany) at 25 °C. Chemical shifts were measured relative to 1 M Na_2_WO_4_ and 85% H_3_PO_4_. ^183^W-NMR samples were prepared in 2.7 mL Na-citrate buffer (50 mM, pH 6.8) with a POT concentration of 2 mM with or without 1 mM *L*-DOPA and measured in 10 mm tubes. The experimental time was ca. 60 h, with a standard pulse program at 20.836 MHz and a 63° flip angle with 1 s relaxation delay. Subsequently, ^31^P-NMR was measured at 202.53 MHz.

### ^183^W-NMR

^183^W-NMR was performed in 50 mM Na-citrate buffer at pH 6.8 to obtain insight into the chemical speciation and hydrolytic stability of POTs. The peaks of the ^183^W-NMR for [**P**_**2**_**W**_**18**_]^6−^ are shifted by approx. 4–5 ppm downfield relative to the assignment in the literature (Table S2). The slight shifting can be explained by different buffer conditions and ionic strength of the solution with regard to the reference data.

The ^183^W-NMR speciation indicates the presence of [*α*_*2*_-P^V^_2_W^VI^_17_O_61_]^10−^ anions in [**P**_**2**_**W**_**18**_]^6−^, [**P**_**2**_**W**_**17**_]^10−^, [**P**_**2**_**W**_**15**_]^12−^ at pH 6.8 (Figs. S8, S12). For the ^183^W-NMR of [**P**_**2**_**W**_**18**_]^6−^ at pH 4 two lines (1:2) were assigned (Fig. S7), whereas for the same compound at neutral pH a mixture of [*α*-P^V^_2_W^VI^_18_O_62_]^6−^ (1:2) and [*α*_*2*_-P^V^_2_W^VI^_17_O_61_]^10−^ (2:2:2:2:1:2:2:2:2) was observed (Fig. S8). Also, the *β*-isomer was detected at pH 4 and pH 6.8, respectively (Figs. S7, S8). Interestingly, the spectra for [**P**_**2**_**W**_**18**_]^6−^ at pH 6.8 with *L*-DOPA exhibits only signals for [*α*_*2*_-P^V^_2_W^VI^_17_O_61_]^10−^ (Fig. S8). Upon storage of [**P**_**2**_**W**_**18**_]^6−^ for 28 d, low amounts of the *α*-isomer were found, but no *β*-isomer was detected (Fig. S12).

### ^31^P-NMR

The presence of [*α*_*2*_-P^V^_2_W^VI^_17_O_61_]^10−^ in the ^183^W-NMR at pH 6.8 was confirmed by two respective signals in the ^31^P-NMR (Figs. 3, S10). The signals at 0 or higher ppm values were assigned to H_*x*_PO_4_^*x*-3^ (*x* = 1, 2), indicating the decomposition of POTs. The signals at − 7.3 ppm and − 14.5 ppm were assigned to [*α*_*2*_-P^V^_2_W^VI^_17_O_61_]^10−^.

### Electrospray ionization mass spectrometry of *AbPPO4*

The mass spectrum (Fig. S2) was recorded with a LTQ Orbitrap Velos Mass spectrometer (Thermo Fisher Scientific, Bremen, Germany) fitted with a nanospray ion source, coupled to a nano HPLC-system (UltiMate 3000, Dionex), followed by deconvolution with Bruker Compass Software.

The sample was loaded on a trap column (µ-Precolumn 5 mm × 300 µ i.d. C4 PepMapp300, 5 µm, 300 Å, Thermo Scientific) with 0.1% trifluoroacetic acid (TFA). The separation of the sample was implemented on a C4 analytical column 50 cm × 75 µm Accucore C4, 2.6 µm, 150 Å (Thermo Fisher Scientific) at a flow rate of 300 nL/min. Mobile Phase A consisted of 2% ACN, 98% H_2_O and 0.1% formic acid (FA). Mobile Phase B comprised 80% ACN, 20% H_2_O and 0.1% FA.

### Dopachrome assay

UV–VIS measurements were carried out on a Shimadzu UV 2401PC following the specific absorption of dopachrome at 475 nm (*ε* = 3700 M^−1^ cm^−1^^7^). For all measurements, 50 mM Na-citrate at pH 6.8 was used as the buffer agent to ensure physiological conditions*.* The stock solutions of POTs were prepared on the day of measurements and used within 3 days.

For a general 1 mL reaction setup in a polystyrene cuvette, 1 µg/mL *Ab*PPO4 (~ 23 nM) was used to catalyze the reaction. The relative inhibition was investigated using 1 mM *L*-DOPA as the substrate and applying an inhibitor concentration range from 0 to 5 mM to obtain activity curves. For the Lineweaver–Burk plots, a *L*-DOPA concentration range of 0.4–1.5 mM was assayed for three different POT concentrations, respectively.

## Supplementary Information

Supplementary Information.
