# Supplementary material for: Wells–Dawson phosphotungstates as mushroom tyrosinase inhibitors: a speciation study
Source: Sci Rep. 2021 Sep 29;11:19354. doi: 10.1038/s41598-021-96491-5 (PMC8481536; doi:10.1038/s41598-021-96491-5)
Supplement: Supplementary file 1 — Supplementary Information. [file 41598_2021_96491_MOESM1_ESM.pdf]

# Supplementary information

## Wells–Dawson phosphotungstates as mushroom tyrosinase inhibitors: a speciation study

Raphael Lampl[1], Joscha Breibeck[1], Nadiia I. Gumerova[1], Mathea Sophia Galanski[2] and Annette Rompel*[1]

*Correspondence to: annette.rompel@univie.ac.at*

[1] Raphael Lampl, MSc., Dr. Joscha Breibeck, Nadiia I. Gumerova, PhD, Univ.-Prof. Dr. Annette Rompel, Universität Wien, Fakultät für Chemie, Institut für Biophysikalische Chemie, Althanstraße 14, 1090 Wien, Austria; http://www.bpc.univie.ac.at.

[2] Ao.Univ.-Prof. Dr. Mathea Sophia Galanski, Universität Wien, Fakultät für Chemie, Institut für Anorganische Chemie und NMR Zentrum, Währinger Straße 42, 1090 Wien, Austria.

**Contents**

1 Structural formulas of *L*-DOPA and kojic acid ........................................................3
2 Biochemical characterization of *Ab*PPO4 ................................................................3
3 Characterization of Wells-Dawson POTs ................................................................5
3.1 IR spectroscopic investigation ........................................................................9
3.2 $^{183}$W-NMR ........................................................................................11
3.3 $^{31}$P-NMR ........................................................................................14
3.4 Long-term stability investigations on $[P_2W_{18}]^{6-}$ by $^{183}$W-NMR and $^{31}$P-NMR ...............16
4 Schemes of structural POT rearrangements ........................................................17
5 Hyperbolic activity curve fit ................................................................................17
6 Curve fit *via* algorithm with Dr-Fit software ..........................................................18
7 Determination of $K_i$- and $\alpha$-parameter through Lineweaver-Burk plot .....................20
8 UV-vis spectroscopic investigation .........................................................................24
9 ESI-MS investigation ............................................................................................24
10 Summary of POT charge densities of Keggin and Wells-Dawson POTs ................25
11 Two isomers of $\alpha$-$[P_2W_{17}]^{10-}$ ........................................................................27
12 Abbreviations .......................................................................................................27
13 References ............................................................................................................28

# 1 Structural formulas of *L*-DOPA and kojic acid

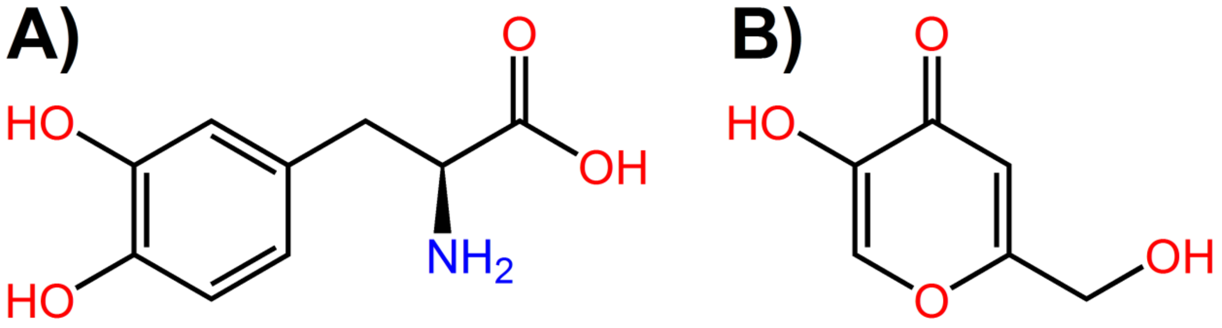


**Figure S1. A)** *L*-3,4-dihydroxyphenylalanine (*L*-DOPA) was used as the substrate for assaying the catechol oxidase activity of *Ab*PPO4.[1] The diphenol group is oxidized to a quinone. *L*-DOPA was added to **[$P_2W_{18}$]**$^{6-}$, **[$P_2W_{17}$]**$^{10-}$, **[$P_2W_{15}$]**$^{12-}$ and **[$P_2W_{12}$]**$^{12-}$ for $^{31}$P-NMR and $^{183}$W-NMR spectroscopic investigation (Figure S9, S11). **B)** The competitive inhibitor kojic acid served as a reference compound with known inhibition characteristics[2] in this study.

# 2 Biochemical characterization of *Ab*PPO4

For ESI-MS analysis, the sample was loaded on a trap column µ-Precolumn 5 mm x 300 µ i.d. C4 PepMapp300, 5 µm, 300 Å (Thermo Scientific) with 0.1% trifluoroacetic acid (TFA). The separation of the sample was implemented on a C4 analytical column 50 cm x 75 µm Accucore C4, 2.6 µm, 150 Å (Thermo Fisher Scientific) at a flow rate of 300 nL/min. Mobile Phase A consisted of 2% ACN, 98% $H_2O$ and 0.1% FA. Mobile Phase B comprised 80% ACN, 20% $H_2O$ and 0.1% FA. The electrospray voltage was set to 2.1 kV and temperature of the ion transfer capillary was 300 °C. The full MS scans were gained in positive ion mode in 400-2000 *m/z* range at a resolution of 7500 (FWHM at 400 *m/z*). The mass obtained for the enzyme was 44269 Da (Figure S2).

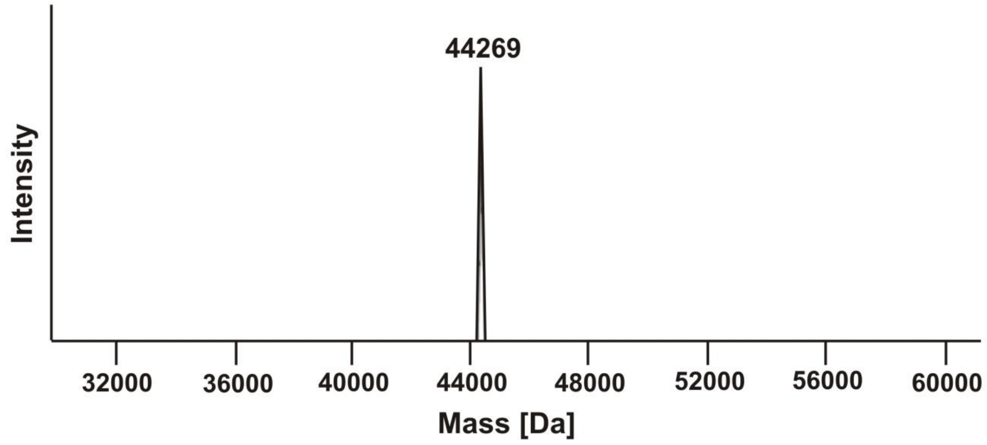


**Figure S2.** ESI-spectrum (after deconvolution) for active *Ab*PPO4 after proteolytic cleavage with Proteinase K.[3]

*Ab*PPO4 contains a vector-derived N-terminal sequence GPLGSPGIP from the protein expression (Figure S3).

GPLGSPGIP

| | | | | | | |
|---|---|---|---|---|---|---|
| MSLLATVGPT | GGVKNRLDIV | DFVRDEKFFT | LYVRALQAIQ | DKDQADYSSF | FQLSGIHGLP | 60 |
| FTPWAKPKDT | PTVPYESGYC | THSQVLFPTW | HRVYVSIYEQ | VLQEAAKGIA | KKFTVHKKEW | 120 |
| AQAAEDLRQP | YWDTGFALVP | PDEIIKLEQV | KITNYDGTKI | TVRNPILRYS | FHPIDPSFNG | 180 |
| YPNFDTWRTT | VRNPDADKKE | NIPALIAKLD | LEADSTREKT | YNMLKFNANW | EAFSNHGEFD | 240 |
| DTHANSLEAV | HDDIHGFVGR | GAIRGHMTHA | LFAAFDPIFW | LHHSNVDRHL | SLWQALYPGV | 300 |
| WVTQGPEREG | SMGFAPGTEL | NKDSALEPFY | ETEDKPWTSV | PLTDTALLNY | SYPDFDKVKG | 360 |
| GTPDLVRDYI | NDHIDRRYGI | KKS | | | | |

**Figure S3.** Protein sequence of *Ab*PPO4 after cutting with Proteinase K; green: main domain of the enzyme to serine (S) 383; violet: histidine (H) at the active center binding the dinuclear copper core; yellow: part of vector sequence.[1]

Through the digestion with Proteinase K the peptide sequence GPLGSP was cut from the main peptide sequence (Table S1). When the ESI-MS was measured under acidic conditions the two copper atoms were lost.

**Table S1.** The mass of *Ab*PPO4 was precisely confirmed by ESI-MS.

| **Theoretical mass of *Ab*PPO4[1] [Da]** | **Mass of vector-derived amino acids GPL [Da]** | **Experimental mass of *Ab*PPO4 [Da]** |
|---|---|---|
| 44536 | - 267 | = 44269 |

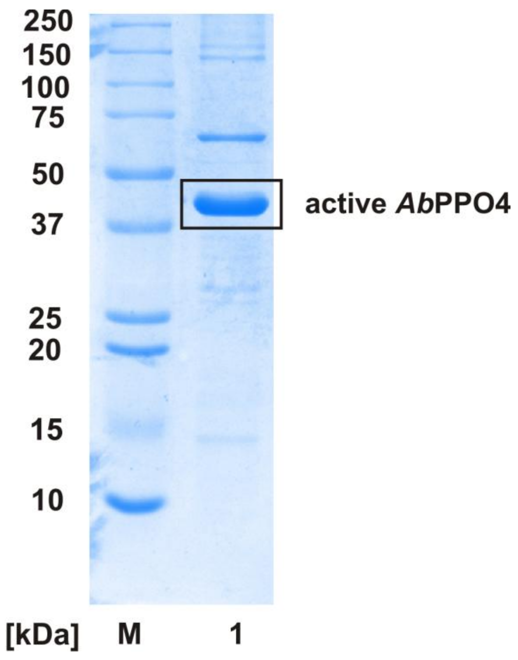


**Figure S4.** SDS-PAGE after chromatographic purification of recombinant *Ab*PPO4 in its active form according to Pretzler *et al.*[1] For preparation of this Figure, the gel was cropped (see Figure S5 for the original image). Lane: M: Precision Plus Protein Standard (BIO-RAD), 1: active *Ab*PPO4: 44.5 kDa.

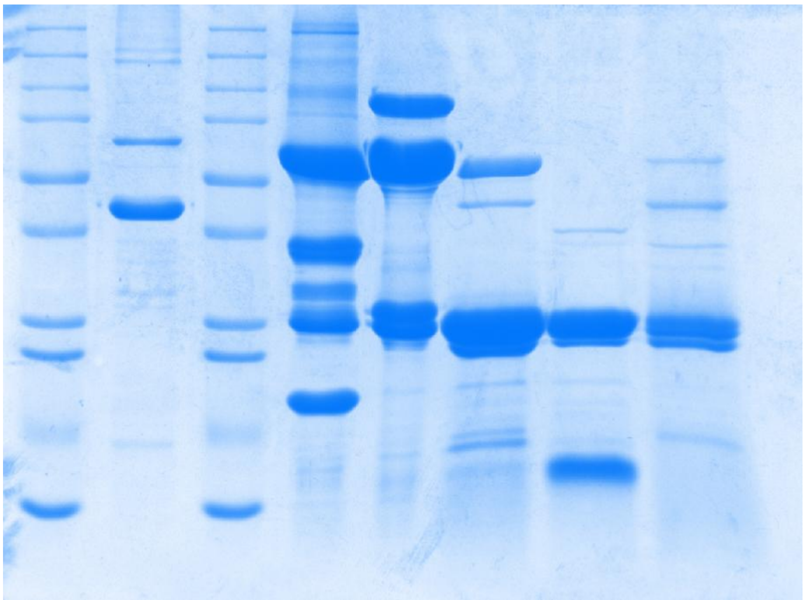

**Figure S5.** Original gel photography of the SDS-PAGE showing all purification steps. The image was taken with a Biorad Molecular Imager Gel Doc. The software ImageLab (version 5.2.1.) was used with standard settings for Coomassie-stained SDS-PAGE.

## 3 Characterization of Wells-Dawson POTs

The synthesized Wells-Dawson POTs were analyzed and characterized by IR as well as $^{183}$W- and $^{31}$P-NMR spectroscopy. The monolacunary Wells-Dawson anion $[P^{V}_{2}W^{VI}_{17}O_{61}]^{10-}$ was verified to be the $\alpha_2$-isomer[4], ($^{183}$W-NMR peak intensities: 2:2:2:2:1:2:2:2:2, $^{31}$P-NMR: two peaks at -7.3 and -13.1 ppm). In [$\mathbf{P_2W_{18}}$]$^{6-}$ at pH 4 and pH 6.8, a mixture of mostly $[\alpha\text{-}P^{V}_{2}W^{VI}_{18}O_{62}]^{6-}$ and $[\beta\text{-}P^{V}_{2}W^{VI}_{18}O_{62}]^{6-}$ ($^{183}$W-NMR peak intensities: 1:1:2:2, $^{31}$P-NMR: two peaks at -11.6 ppm and -12.4 ppm) was revealed. The $\beta$-isomer is a common byproduct during the synthesis of the $\alpha$-isomer.[5]

**Table S2.** Wells-Dawson POTs synthesized and tested for inhibitory activity against *Ab*PPO4. Numbered superscripts in round brackets refer to the assignment of NMR signals to POT species in solution: (1) $[\alpha\text{-}P^{V}_{2}W^{VI}_{18}O_{62}]^{6-}$, (2) $[\beta\text{-}P^{V}_{2}W^{VI}_{18}O_{62}]^{6-}$, (3) $[\alpha_{2}\text{-}P^{V}_{2}W^{VI}_{17}O_{61}]^{10-}$, (4) $[H_{2}P^{V}_{2}W^{VI}_{12}O_{48}]^{12-}$, (5) $[P^{V}_{8}W^{VI}_{48}O_{184}]^{40-}$, (6) $[P^{V}_{2}W^{VI}_{19}O_{69}(H_{2}O)]^{14-}$. The table shows: [a] solution pH for NMR spectroscopy, [b] time between solution preparation and NMR measurement, [c] POT crystal structure first reported by, [d] POT synthesis according to, [e] assignment according to, [f] assignment according to.

| **Wells-Dawson POT** | **Abbreviation when applied to solution** | **pH [a]** | **Time [b]** | **$^{183}$W-NMR ($\delta_W$) [ppm]** | **$^{31}$P-NMR ($\delta_P$) [ppm]** | **Reference** | | | |
|---|---|---|---|---|---|---|---|---|---|
| | | | | | | **First reported in [c]** | **Synthesis [d]** | **$^{31}$P NMR [e]** | **$^{183}$W NMR [f]** |
| $K_6[\alpha^{(1)}/\beta^{(2)}\text{-}P^{V}_{2}W^{VI}_{18}O_{62}]\cdot 14\ H_2O$ | **$[P_2W_{18}]^{6-}$** | 6.8 | 1 d | -107.3$^{(2)}$, -123.6$^{(1)}$, -125.4$^{(3)}$, -129.9$^{(2)}$, -137.8$^{(3)}$, -158.4$^{(3)}$, -166.9$^{(2)}$, -168.8$^{(1)}$, -172.9$^{(3)}$, -178.1$^{(3)}$, -186.4$^{(2)}$, -216.5$^{(3)}$, -218.9$^{(3)}$, -221.3$^{(3)}$, -239.4$^{3)}$ | 2.3, 0.0, -7.3$^{(3)}$, -8.3, -11.6$^{(2)}$, -12.4$^{(2)}$, -13.1$^{(1)}$, -14.5$^{(3)}$ | 6 | 5* | 5 | 7 |
| $K_6[\alpha^{(1)}/\beta^{(2)}\text{-}P^{V}_{2}W^{VI}_{18}O_{62}]\cdot 14\ H_2O^{(2)}$ | **$[P_2W_{18}]^{6-}$** | 4 | 1 d | -107.4$^{(2)}$, -123.6$^{(1)}$, -127.1$^{(2)}$, -166.9$^{(2)}$, -168.8$^{(1)}$, -186.3$^{(2)}$ | -11.6$^{(2)}$, -12.4$^{(2)}$, -13.1$^{(1)}$ | 6 | 5* | 5 | 7 |
| $(NH_4)_6[\alpha^{(1)}/\beta^{(2)}\text{-}P^{V}_{2}W^{VI}_{18}O_{62}]\cdot 14\ H_2O$ + *L*-DOPA | **$[P_2W_{18}]^{6-}$** + *L*-DOPA | 6.8 | 1 d | -125.5$^{(3)}$, -138.0$^{(3)}$, -158.4$^{(3)}$, -173.0$^{(3)}$, -178.3$^{(3)}$, -216.9$^{(3)}$, -219.6$^{(3)}$, -222.0$^{(3)}$, -239.9$^{(3)}$ | 2.7, 0.2, -7.3$^{(3)}$, -7.5, -8.1, -9.5, -14.5$^{(3)}$ | 6 | 5* | 5 | 7 |
| $K_6[\alpha^{(1)}/\beta^{(2)}\text{-}P^{V}_{2}W^{VI}_{18}O_{62}]\cdot 14\ H_2O$ | **$[P_2W_{18}]^{6-}$** | 6.8 | 28 d | -86.4, -123.8$^{(1)}$, -125.3$^{(3)}$, -137.4$^{(3)}$, | 2.4, 0.0, -7.4$^{(3)}$, -7.5, | 6 | 5* | 5 | 7 |

| | | | | | | | | | |
|---|---|---|---|---|---|---|---|---|---|
| | | | | -158.6(3), -169.1(1), -173.3(3), -177.8(3), -215.7(3), -218.4(3), -220.5(3), -238.6(3) | -8.3, -8.6, -10.0, -13.1, -14.0, -14.4(3) | | | | |
| $K_{10}[\alpha_2\text{-}P^{V}_2W^{VI}_{17}O_{61}]^{(3)}\cdot 20\ H_2O$ | **$[P_2W_{17}]^{10-}$** | 6.8 | 1 d | -125.0(3), -137.5(3), -157.6(3), -172.1(3), -177.7(3), -216.0(3), -218.9(3), -221.2(3), -238.8 (3) | -7.3(3), -7.8, -8.8, -9.4, -9.9, -14.5(3) | 8 | 5 | 5 | 7 |
| $K_{10}[\alpha_2\text{-}P^{V}_2W^{VI}_{17}O_{61}]^{(3)}\cdot 20\ H_2O$ | **$[P_2W_{17}]^{10-}$** + *L*-DOPA | 6.8 | 1 d | (not performed due to the species determination by $^{31}P$-NMR) | -7.3(3), -7.8, -8.8, -9.4, -9.9, -14.5(3) | 8 | 5 | 5 | / |
| $K_{12}[\alpha\text{-}P^{V}_2W^{VI}_{15}O_{56}]\cdot 24\ H_2O$ | **$[P_2W_{15}]^{12-}$** | 6.8 | 1 d | -125.0(3), -137.5(3), -157.6(3), -172.1(3), -177.7(3), -216.1(3), -218.9 (3), -222.7(3), -238.7 (3) | 3.0, 1.0, -7.3(3), -9.4, -14.5(3) | 9 | 5 | 5 | 7 |
| $K_{12}[\alpha\text{-}P^{V}_2W^{VI}_{15}O_{56}]\cdot 24\ H_2O$ + *L*-DOPA | **$[P_2W_{15}]^{12-}$** + *L*-DOPA | 6.8 | 1 d | (not performed due to the species determination by $^{31}P$-NMR) | 4.4, 2.4, -7.3(3), -8.0, -14.4(3) | 9 | 5 | 5 | / |
| $(NH_4)_{12}[\alpha\text{-}H_2P^{V}_2W^{VI}_{12}O_{48}]\cdot 24\ H_2O$ | **$[P_2W_{12}]^{12-}$** | 6.8 | 1 d | -190.3(5), -191.6(5), -211.3(5) | 4.5, 4.0, 1.7, -7.3, -7.5(5), -8.3(4), -9.3, -9.5(6), -14.4 | 10 | 5 | 5, 11, 12 | 13 |

| | | | | | | | | | |
|---|---|---|---|---|---|---|---|---|---|
| $(NH_4)_{12}$[*α*-$H_2P^{V}_2W^{VI}_{12}O_{48}$]·24 $H_2O$ + *L*-DOPA | [$\mathbf{P_2W_{12}}$]$^{12-}$ + *L*-DOPA | 6.8 | 1 d | (not performed due to the species determination by $^{31}$P-NMR) | 4.6, 3.9, 1.8, -7.3, -7.4$^{(5)}$, -8.3$^{(4)}$, -9.2, -9.5$^{(6)}$, -14.4 | 10 | 5 | 5, 11, 12 | / |

* The procedure leads to the synthesis of alpha and beta isomers [5]:

In a 2-L beaker a sample of 250 g (0.76 mol) of $Na_2WO_4$·$2H_2O$ is dissolved in 500 mL of water and 210 mL (3.09 mol) of orthophosphoric acid (85%) is added. The solution is heated at reflux for 4 h. After cooling, 100 g of ammonium chloride is added, and the solution is stirred for 10 min. The crude ammonium salt obtained from the refluxed solution is washed by stirring in a solution of 25 g of $NH_4Cl$ in 100 mL of water for 10 min and is then redissolved in 250 mL of warm water (~ 45 $^{o}$C). Potassium chloride (40 g) is added to the cold solution. The potassium salt is removed by filtering and is then dissolved in 250 mL of hot water (~ 80 $^{o}$C). The white needles of triacontatungstopentaphosphate that appear upon cooling to 15 $^{o}$C are removed by filtration. The filtrate is either treated directly with 25 g of potassium chloride to get a mixture of the two potassium octadecatungstodiphosphate isomers, or it is refluxed for 6 h to get a solution of the pure *A* isomer, that is then precipitated with 25 g of potassium chloride. In either case the precipitate is filtered on a sintered glass frit and air dried for 3 days.

## 3.1 IR spectroscopic investigation

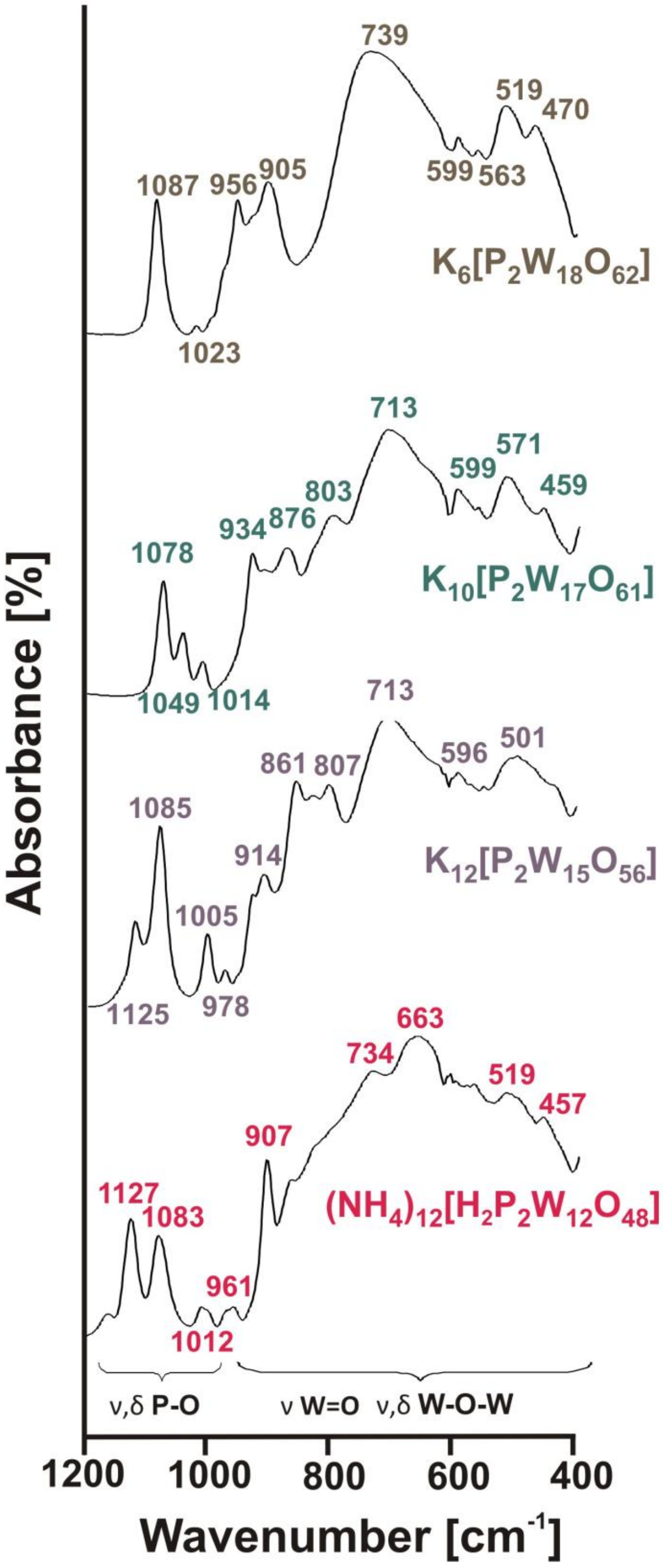


**Figure S6.** IR spectra of the Wells-Dawson POTs in the region of W-O-W, W-O-P and W=O bond vibrations (1200 – 400 $cm^{-1}$).

**Table S3:** Comparison of experimental and reported[5] wavenumbers for P–O vibrations and $^{31}P$ NMR shifts

| **POT** | **IR spectroscopy** | | **$^{31}P$ NMR spectroscopy** | |
|---|---|---|---|---|
| | **Experimental (Figure S6)** | **Reported in [5]** | **Experimental (Figure S10)** | **Reported in [5]** |
| $K_6[\alpha/\beta\text{-}P^{V}_2W^{VI}_{18}O_{62}]\cdot 14H_2O$ | 1087 and 1023 $cm^{-1}$ | 1087 and 1023 $cm^{-1}$ | -13.1 ppm for *α* isomer and -11.6 and -12.4 ppm for *β* isomer | -12.5 ppm for *α* isomer and -11.0 and -11.7 ppm for *β* isomer |
| $K_{10}[\alpha_2\text{-}P^{V}_2W^{VI}_{17}O_{61}]\cdot 20H_2O$ | 1078, 1049 and 1014 $cm^{-1}$ | 1084, 1050 and 1012 $cm^{-1}$ | -7.2 and -14.4 ppm | -7.1 and -13.6 ppm |
| $K_{12}[P^{V}_2W^{VI}_{15}O_{56}]\cdot 24H_2O$ | 1125, 1085 and 1005 $cm^{-1}$ | 1130, 1086 and 1009 $cm^{-1}$ | due to low solubility and fast rearrangement to $[\alpha_2\text{-}P^{V}_2W^{VI}_{17}O_{61}]^{10-}$ no spectrum could be recorded | +0.1 and -13.3 ppm |
| $(NH_4)_{12}[H_2P^{V}_2W^{VI}_{12}O_{48}]\cdot 24H_2O$ | 1127, 1083 and 1012 $cm^{-1}$ | 1130, 1075 and 1012 $cm^{-1}$ | -8.6 ppm | -8.6 ppm |

## 3.2 $^{183}$W-NMR

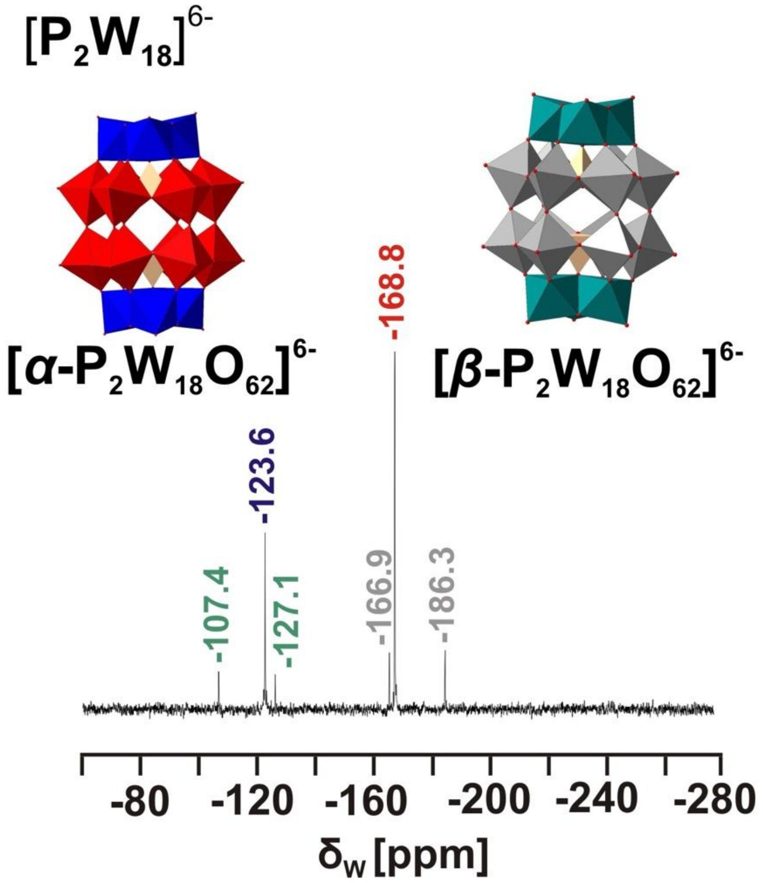


**Figure S7.** $^{183}$W-NMR of [$\mathbf{P_2W_{18}}$]$^{6-}$ in water at pH 4. For signal assignments see Table S2. *Color code*: [$\alpha$-$P^V_2W^{VI}_{18}O_{62}$]$^{6-}$: cap of {$WO_6$} octahedra, blue; belt of {$WO_6$} octahedra, red; [$\beta$-$P^V_2W^{VI}_{18}O_{62}$]$^{6-}$: cap of {$WO_6$} octahedra, petrol; belt of {$WO_6$} octahedra, grey; {$PO_4$} tetrahedron, tan.

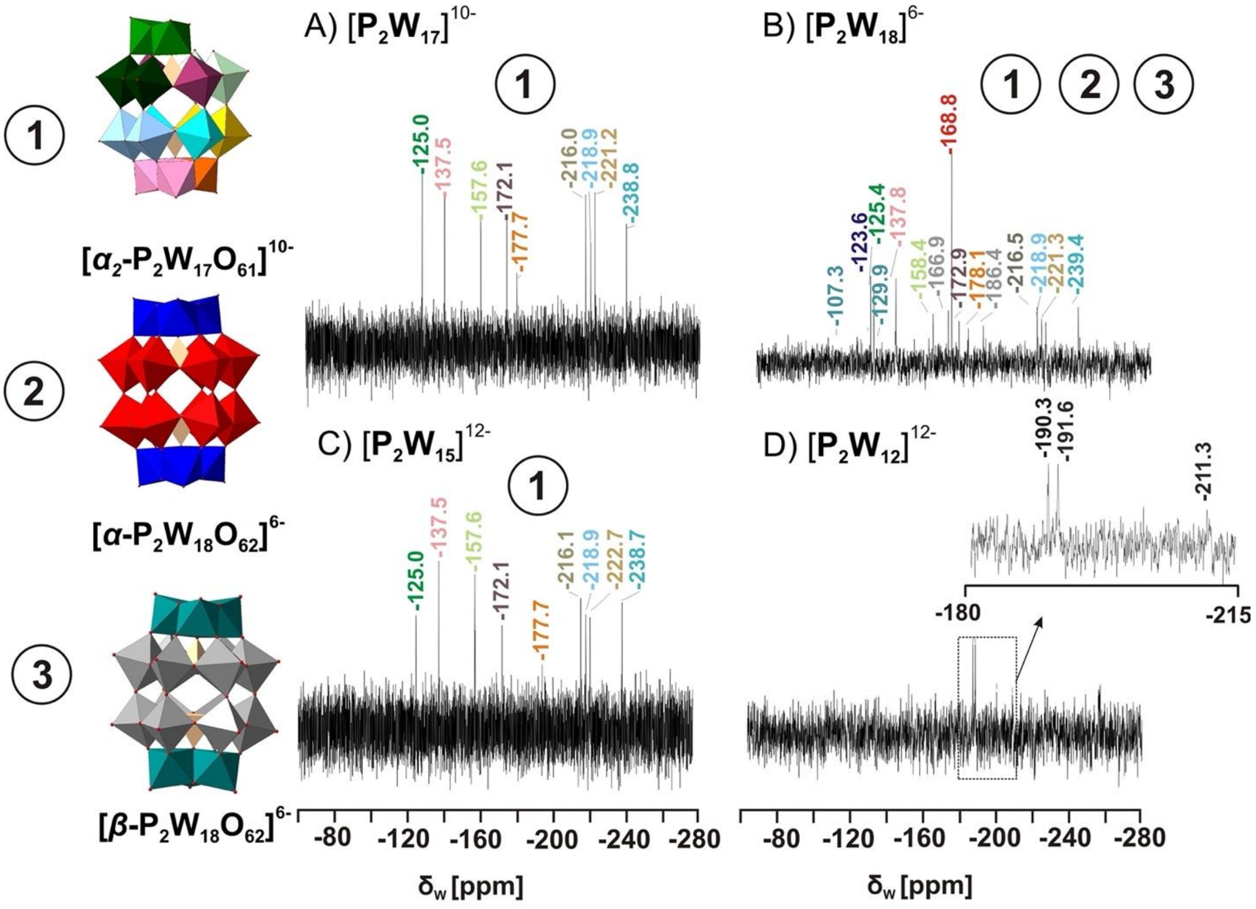


**Figure S8.** $^{183}$W-NMR spectra of [$\mathbf{P_2W_{18}}$]$^{6-}$, [$\mathbf{P_2W_{17}}$]$^{10-}$, [$\mathbf{P_2W_{15}}$]$^{12-}$ and [$\mathbf{P_2W_{12}}$]$^{12-}$ in 50 mM Na-citrate buffer at pH 6.8: A) educt/product: [$\alpha_2$-$P^V{}_2W^{VI}{}_{17}O_{61}$]$^{10-}$; B) educts: [$\alpha$-$P^V{}_2W^{VI}{}_{18}O_{62}$]$^{6-}$ and [$\beta$-$P^V{}_2W^{VI}{}_{18}O_{62}$]$^{6-}$, products: [$\alpha$-$P^V{}_2W^{VI}{}_{18}O_{62}$]$^{6-}$, [$\beta$-$P^V{}_2W^{VI}{}_{18}O_{62}$]$^{6-}$ and [$\alpha_2$-$P^V{}_2W^{VI}{}_{17}O_{61}$]$^{10-}$; C) educt: [$P^V{}_2W^{VI}{}_{15}O_{56}$]$^{12-}$; product: [$\alpha_2$-$P^V{}_2W^{VI}{}_{17}O_{61}$]$^{10-}$; D) educt: [$H_2P^V{}_2W^{VI}{}_{12}O_{48}$]$^{12-}$; product: [$P^V{}_8W^{VI}{}_{48}O_{184}$]$^{40-}$. For signal assignments see Table S2. *Color code*: [$\alpha_2$-$P^V{}_2W^{VI}{}_{17}O_{61}$]$^{10-}$ {$WO_6$} octahedra: top, olive green; upper belt left, dark grey; upper belt middle, lavender; upper belt right, light green; lower belt left, light blue; lower belt middle, turquoise; lower belt right, gold; bottom left, pink; bottom right, orange; [$\alpha$-$P^V{}_2W^{VI}{}_{18}O_{62}$]$^{6-}$: cap of {$WO_6$} octahedra, blue; belt of {$WO_6$} octahedra, red; [$\beta$-$P^V{}_2W^{VI}{}_{18}O_{62}$]$^{6-}$: cap of {$WO_6$} octahedra, petrol; belt of {$WO_6$} octahedra, grey; {$PO_4$} tetrahedra, tan.

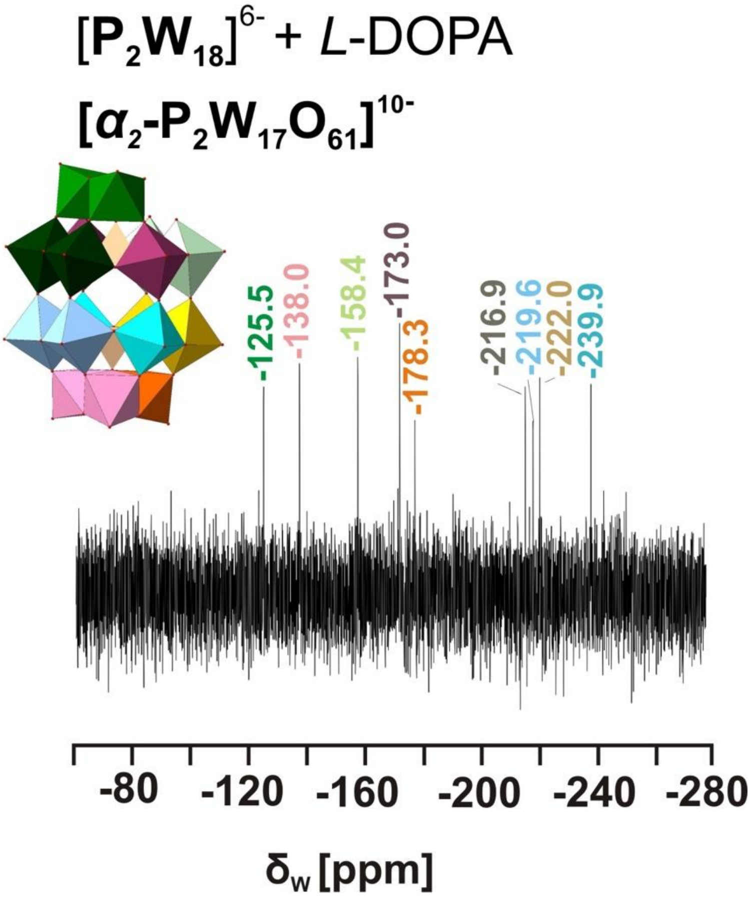


**Figure S9.** $^{183}$W-NMR spectrum of [**$P_2W_{18}$**]$^{6-}$. Rearrangement to [$\alpha_2$-$P^V{}_2W^{VI}{}_{17}O_{61}$]$^{10-}$ was observed. For signal assignments see Table S2. *Color code*: [$\alpha_2$-$P^V{}_2W^{VI}{}_{17}O_{61}$]$^{10-}$ {$WO_6$} octahedra: A) top, olive green; upper belt left, dark grey; upper belt middle, lavender; upper belt right, light green; lower belt left, light blue; lower belt middle, turquoise; lower belt right, gold; bottom left, pink; bottom right, orange; {$PO_4$} tetrahedra, tan.

## 3.3 $^{31}$P-NMR

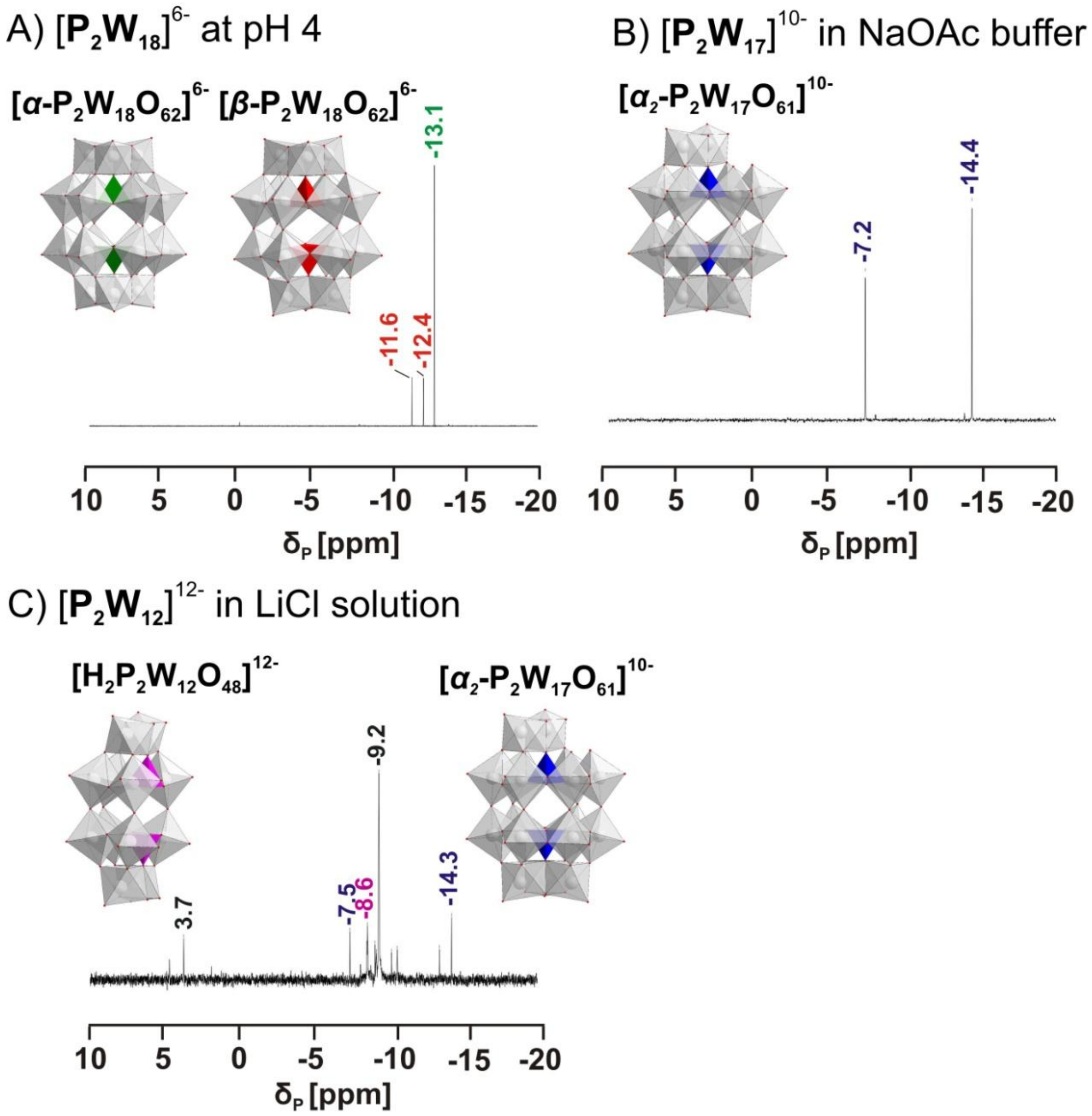


**Figure S10.** $^{31}$P-NMR of A) [$\mathbf{P_2W_{18}}$]$^{6-}$ at pH 4; B) [$\mathbf{P_2W_{17}}$]$^{10-}$ in Na-acetate buffer pH 5.6; C) [$\mathbf{P_2W_{12}}$]$^{12-}$ in 1 M LiCl solution pH 6.2. For signal assignments see Table S2. A) [$\boldsymbol{\alpha}$-$\mathbf{P_2W_{18}}$]$^{6-}$ and [$\boldsymbol{\beta}$-$\mathbf{P_2W_{18}}$]$^{6-}$ were verified by the literature (ref. [3]). B) The purity of synthesized $K_{10}[\alpha_2\text{-}P^V{}_2W^{VI}{}_{17}O_{61}]\cdot 20H_2O$ has been confirmed with two signals originating from $[\alpha_2\text{-}P^V{}_2W^{VI}{}_{17}O_{61}]^{10-}$; C) Although the hydrolysis of $(NH_4)_{12}[H_2P^V{}_2W^{VI}{}_{12}O_{48}]\cdot 24H_2O$ is pronounced in LiCl solution, the signal at -8.6 ppm, which corresponds to the hexalacunary anion, is present. The most intense signal at -9.2 ppm can be assigned to $[P^V{}_2W^{VI}{}_{19}O_{69}(H_2O)]^{14-}$. Color code*:* {$WO_6$} octahedra, grey; {$PO_4$} tetrahedra in $[\alpha\text{-}P^V{}_2W^{VI}{}_{18}O_{62}]^{6-}$, green; {$PO_4$} tetrahedra in $[\beta\text{-}P^V{}_2W^{VI}{}_{18}O_{62}]^{6-}$, red; {$PO_4$} tetrahedra in $[\alpha_2\text{-}P^V{}_2W^{VI}{}_{17}O_{61}]^{10-}$, blue; {$PO_4$} tetrahedra in $[H_2P^V{}_2W^{VI}{}_{12}O_{48}]^{12-}$, pink.

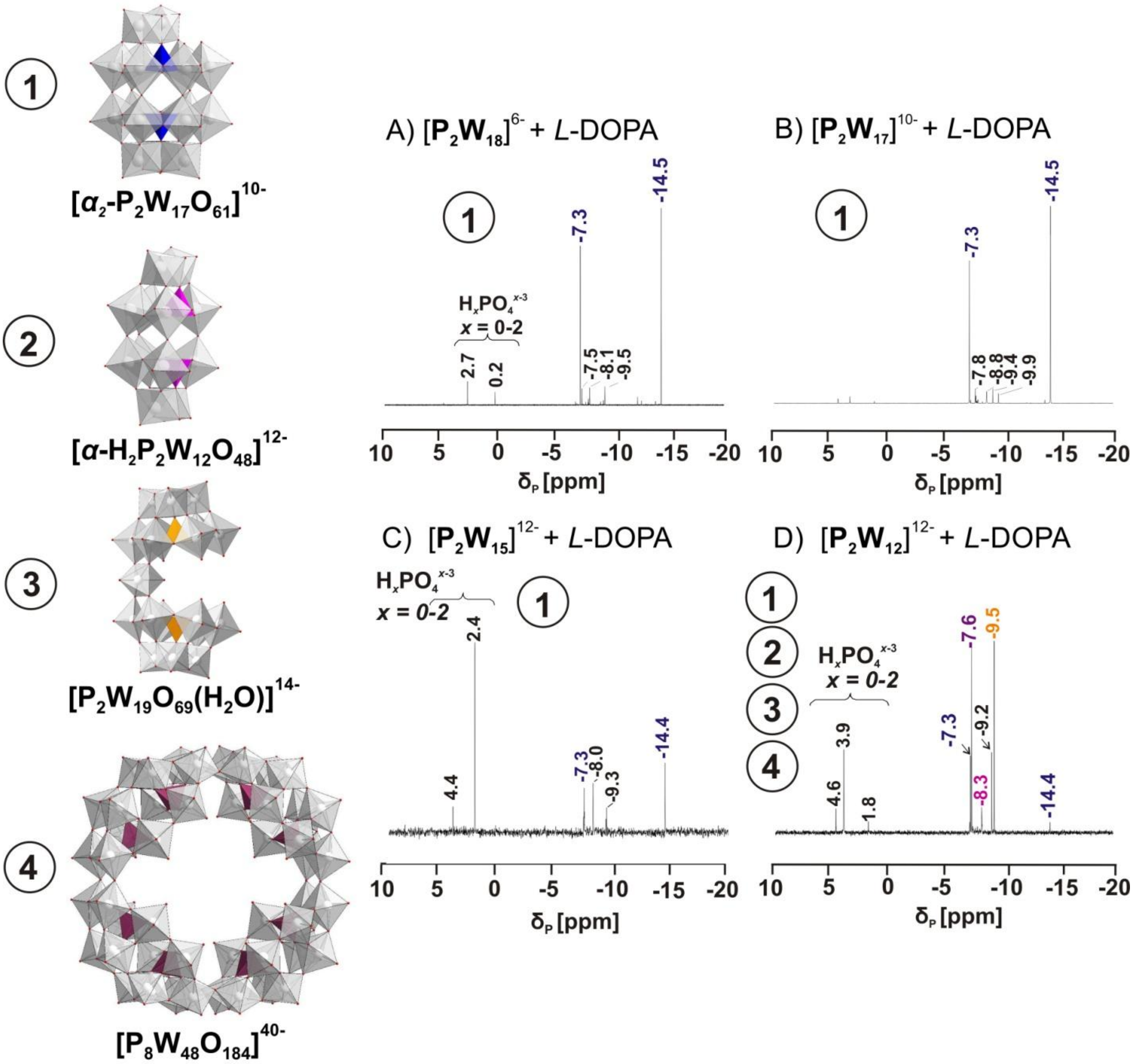


**Figure S11.** $^{31}$P-NMR of A) $[\mathbf{P_2W_{18}}]^{6-}$ + *L*-DOPA; B) $[\mathbf{P_2W_{17}}]^{10-}$ + *L*-DOPA; C) $[\mathbf{P_2W_{15}}]^{12-}$ + *L*-DOPA; D) $[\mathbf{P_2W_{12}}]^{12-}$ + *L*-DOPA. A) educts: $[\alpha\text{-}P^{V}_2W^{VI}_{18}O_{62}]^{6-}$ and $[\beta\text{-}P^{V}_2W^{VI}_{18}O_{62}]^{6-}$; product: $[\alpha_2\text{-}P^{V}_2W^{VI}_{17}O_{61}]^{10-}$; B) educt $[\alpha_2\text{-}P^{V}_2W^{VI}_{17}O_{61}]^{10-}$; product: $[\alpha_2\text{-}P^{V}_2W^{VI}_{17}O_{61}]^{10-}$; C) educt: $[P^{V}_2W^{VI}_{15}O_{56}]^{12-}$; product: $[\alpha_2\text{-}P^{V}_2W^{VI}_{17}O_{61}]^{10-}$; D) educt: $[H_2P^{V}_2W^{VI}_{12}O_{48}]^{12-}$; products: $[P^{V}_8W^{VI}_{48}O_{184}]^{40-}$, $[P^{V}_2W^{VI}_{19}O_{69}(H_2O)]^{14-}$, $[H_2P^{V}_2W^{VI}_{12}O_{48}]^{12-}$, $[\alpha_2\text{-}P^{V}_2W^{VI}_{17}O_{61}]^{10-}$. Color code: $\{WO_6\}$ octahedra, grey; $\{PO_4\}$ tetrahedra blue, pink, orange, burgundy.

### 3.4 Long-term stability investigations on $[\mathbf{P_2W_{18}}]^{6-}$ by $^{183}$W-NMR and $^{31}$P-NMR

To explore the long-term stability of Wells-Dawson POTs, the sample composition was analyzed after 4 weeks of storage in Na-citrate buffer (pH 6.8) at room temperature. $^{183}$W-NMR and $^{31}$P-NMR investigations were carried out for $[\mathbf{P_2W_{18}}]^{6-}$ at pH 6.8. After 28 days, a concentration decrease of $[\alpha\text{-}P^{V}{}_2W^{VI}{}_{18}O_{62}]^{6-}$ as well as the disappearance of $[\beta\text{-}P^{V}{}_2W^{VI}{}_{18}O_{62}]^{6-}$ were observed. Furthermore, a slight increase in the phosphate peaks at 0.0 and 2.4 ppm (Figure S13) was detected, which indicated decomposition of $[\alpha/\beta\text{-}P^{V}{}_2W^{VI}{}_{18}O_{62}]^{6-}$.

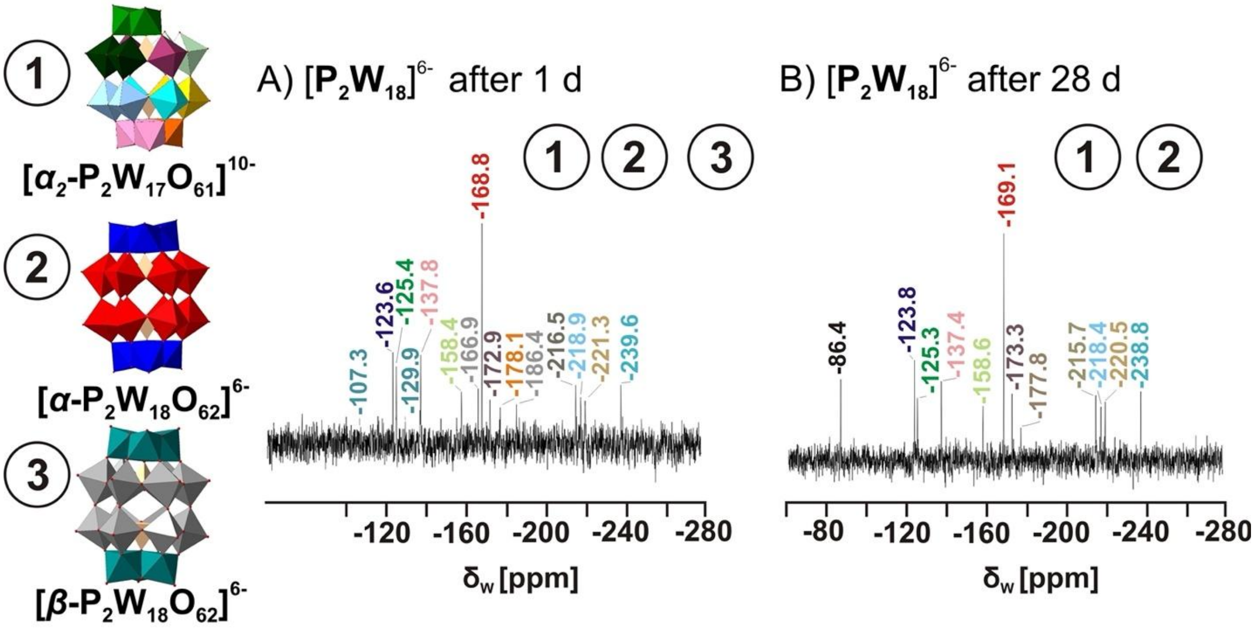


**Figure S12.** $^{183}$W-NMR spectra of $[\mathbf{P_2W_{18}}]^{6-}$ after 1 d (A) and 28 d (B) in 50 mM Na-citrate buffer at pH 6.8: A) educts: $[\alpha\text{-}P^{V}{}_2W^{VI}{}_{18}O_{62}]^{6-}$ and $[\beta\text{-}P^{V}{}_2W^{VI}{}_{18}O_{62}]^{6-}$, products: $[\alpha\text{-}P^{V}{}_2W^{VI}{}_{18}O_{62}]^{6-}$, $[\beta\text{-}P^{V}{}_2W^{VI}{}_{18}O_{62}]^{6-}$ and $[\alpha_2\text{-}P^{V}{}_2W^{VI}{}_{17}O_{61}]^{10-}$. B) educts: $[\alpha\text{-}P^{V}{}_2W^{VI}{}_{18}O_{62}]^{6-}$ and $[\beta\text{-}P^{V}{}_2W^{VI}{}_{18}O_{62}]^{6-}$; products: $[\alpha\text{-}P^{V}{}_2W^{VI}{}_{18}O_{62}]^{6-}$ and $[\alpha_2\text{-}P^{V}{}_2W^{VI}{}_{17}O_{61}]^{10-}$. For signal assignments see Table S2. *Color code*: $[\alpha_2\text{-}P^{V}{}_2W^{VI}{}_{17}O_{61}]^{10-}$ $\{WO_6\}$ octahedra: top, olive green; upper belt left, dark grey; upper belt middle, lavender; upper belt right, light green; lower belt left, light blue; lower belt middle, turquoise; lower belt right, gold; bottom left, pink; bottom right, orange; $[\alpha\text{-}P^{V}{}_2W^{VI}{}_{18}O_{62}]^{6-}$: cap of $\{WO_6\}$ octahedra, blue; belt of $\{WO_6\}$ octahedra, red; $[\beta\text{-}P^{V}{}_2W^{VI}{}_{18}O_{62}]^{6-}$: cap of $\{WO_6\}$ octahedra, petrol; belt of $\{WO_6\}$ octahedra, grey; $\{PO_4\}$ tetrahedra, tan.

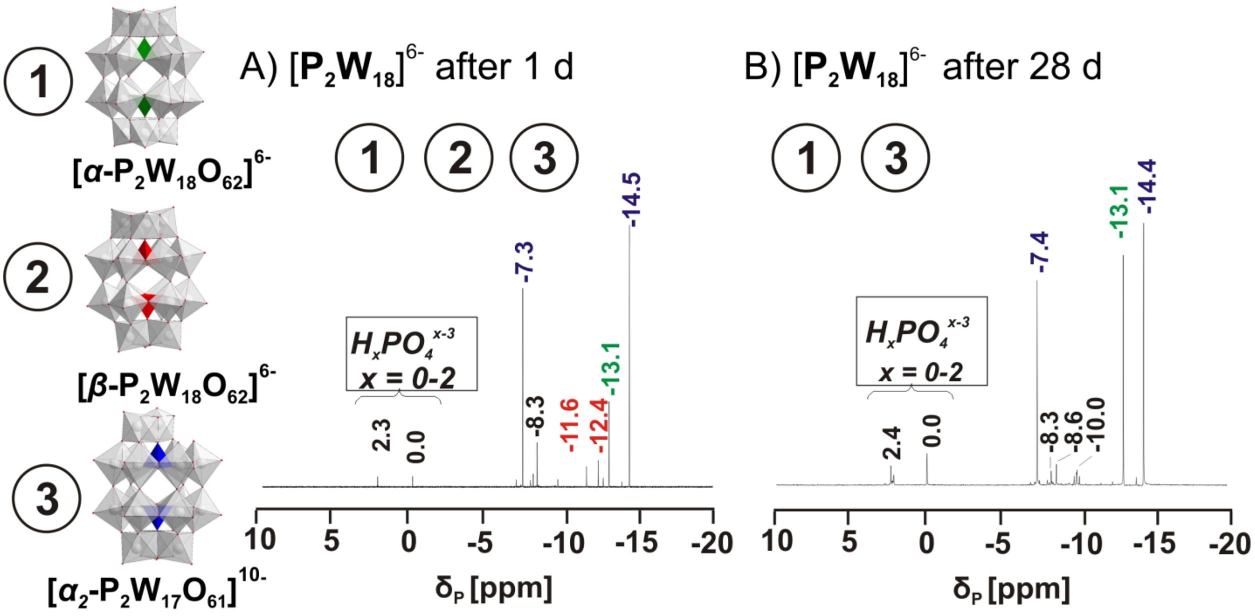


**Figure S13.** $^{31}$P-NMR spectra of $[\mathbf{P_2W_{18}}]^{6-}$ after 1 d (A) and 28 d (B) in 50 mM Na-citrate buffer at pH 6.8: A) educts: $[\alpha\text{-}P^V{}_2W^{VI}{}_{18}O_{62}]^{6-}$ and $[\beta\text{-}P^V{}_2W^{VI}{}_{18}O_{62}]^{6-}$; products: $[\alpha\text{-}P^V{}_2W^{VI}{}_{18}O_{62}]^{6-}$, $[\beta\text{-}P^V{}_2W^{VI}{}_{18}O_{62}]^{6-}$ and $[\alpha_2\text{-}P^V{}_2W^{VI}{}_{17}O_{61}]^{10-}$. B) educts: $[\alpha\text{-}P^V{}_2W^{VI}{}_{18}O_{62}]^{6-}$ and $[\beta\text{-}P^V{}_2W^{VI}{}_{18}O_{62}]^{6-}$; products: $[\alpha\text{-}P^V{}_2W^{VI}{}_{18}O_{62}]^{6-}$ and $[\alpha_2\text{-}P^V{}_2W^{VI}{}_{17}O_{61}]^{10-}$. In comparison to the measurement after 1 d, an extinction of signals for $[\beta\text{-}P^V{}_2W^{VI}{}_{18}O_{62}]^{6-}$ was discovered, demonstrating complete conversion to the *α*-form. For signal assignments see Table S2. *Color code:* {$WO_6$} octahedra, grey; {$PO_4$} tetrahedra in $[\alpha\text{-}P^V{}_2W^{VI}{}_{18}O_{62}]^{6-}$, green; {$PO_4$} tetrahedra in $[\beta\text{-}P^V{}_2W^{VI}{}_{18}O_{62}]^{6-}$, red; {$PO_4$} tetrahedra in $[\alpha_2\text{-}P^V{}_2W^{VI}{}_{17}O_{61}]^{10-}$, blue.

## 4 Schemes of structural POT rearrangements

The rearrangement processes at pH 6.8 for the Wells-Dawson POTs under study identified by $^{31}$P- and $^{183}$W-NMR analysis in the preceding sections are illustrated in Scheme S1:

$$17\ [P_2W_{18}O_{62}]^{6-} + 2\ HPO_4^{2-} + 74\ OH^- \longrightarrow 18\ [P_2W_{17}O_{61}]^{10-} + 38\ H_2O \qquad (I)$$

$$17\ [P_2W_{15}O_{56}]^{12-} + 46\ H^+ \longrightarrow 15\ [P_2W_{17}O_{61}]^{10-} + 4\ HPO_4^{2-} + 21\ H_2O \qquad (II)$$

$$17\ [H_2P_2W_{12}O_{48}]^{12-} + 64\ H^+ \longrightarrow 12\ [P_2W_{17}O_{61}]^{10-} + 10\ HPO_4^{2-} + 44\ H_2O \qquad (III)$$

$$4\ [H_2P_2W_{12}O_{48}]^{12-} + 8\ H^+ \longrightarrow [P_8W_{48}O_{184}]^{40-} + 8\ H_2O \qquad (IV)$$

$$19\ [H_2P_2W_{12}O_{48}]^{12-} + 32\ H^+ \longrightarrow 12\ [P_2W_{19}O_{69}(H_2O)]^{14-} + 14\ HPO_4^{2-} + 16\ H_2O \qquad (V)$$

**Scheme S1:** Proposed chemical equations for the structural interconversions observed in buffered solutions of Wells-Dawson POTs at pH 6.8. $HPO_4^{2-}$ was chosen as the dominant phosphate form at neutral pH, and other protonation states of POT anions were neglected for simplification. Note that the degree of protonation does not affect the structural scaffolds illustrated here.

## 5 Hyperbolic activity curve fit

Copeland *et al.* [14] presented the hyperbolic curve fit for enzymatic activity based on the Michaelis-Menten equation (1), which is

$$v = v_{max} \frac{[S]}{K_M + [S]}. \qquad (1)$$

$v$ is the enzymatic reaction velocity, $v_{max}$ the maximal enzymatic velocity of the enzyme, $K_M$ the concentration for the half-maximal velocity and $[S]$ the substrate concentration. If enzyme inhibition is taken into account, apparent values (e.g., $v_{app}$, $v_{max,app}$, $K_{M,app}$) are employed. For all inhibition mechanisms the apparent maximal velocity $v_{max,app}$ is given by

$$v_{max,app} = \frac{v_{max}}{1 + \frac{[I]}{\alpha K_i}} \tag{2}$$

and the apparent Michalis-Menten constant $K_{M,app}$ is

$$K_{M,app} = \frac{K_M(1 + \frac{[I]}{K_i})}{1 + \frac{[I]}{\alpha K_i}} \tag{3}$$

Equations (3) and (2) are inserted into equation (1). To obtain the relative inhibition (in %), $v_{app}$ is divided by the uninhibited enzymatic velocity $v$.

$$\frac{v_{app}}{v} = 100\frac{K_M + [S]}{K_M\left(1 + \frac{[I]}{K_i}\right) + [S]\left(1 + \frac{[I]}{\alpha K_i}\right)} \tag{4}$$

Herein, *[I]* refers to the inhibitor concentration, $K_i$ is the inhibition constant measuring the inhibitory capacity and the parameter $\alpha$ gives information on the exhibited mode of inhibition. The three ideal inhibition types are reflected by three limiting conditions for $\alpha$, with $\alpha$ as quotient of the inhibition constant of the uncompetitive and competitive inhibition:

(A) $0 < \alpha \ll 1$: uncompetitive inhibition

(B) $\alpha = 1$: non-competitive inhibition

(C) $\alpha \gg 1$: competitive inhibition

Therefore, values of $\alpha < 1$ often correspond to mixed-type inhibition with contributions from uncompetitive and non-competitive inhibition modes.

The concentration of *L*-DOPA was kept constant at 1 mM for all experiments. As determined by Pretzler *et al.*[1], $K_M$ was set to 26.1 mM. The final hyperbolic equation (5) was used for the curve fit with OriginPro 8:

$$\frac{v_{app}}{v} = 100\frac{27.1}{27.1 + \left(\frac{26.1 + \frac{1}{\alpha}}{K_i}\right)[I]}. \tag{5}$$

The initial parameters for the regression were taken from the Lineweaver-Burk plots as described in Section 7.

## 6 Curve fit *via* algorithm with Dr-Fit software

To investigate the underlying inhibition processes in more detail (e.g., multiple stimulatory or inhibitory effects), a multiphasic model was employed to fit the data. Therefore, an algorithm

implemented in the Dr-Fit software (https://sourceforge.net/projects/drfit/) was used for modeling[15]. The basic mathematical entity of the program is the Hill model based on equation

$$E_{Hill}(C; E_\infty, E_0, EC_{50}, H) = E_0 + \frac{E_\infty - E_0}{1 + \left(\frac{EC_{50}}{C}\right)^H}. \tag{6}$$

The $EC_{50}$ value is the concentration at half-maximal effect, $E_0$ is the effect if no inhibitor is present, $H$ is the Hill exponent, $E_\infty$ is the maximal effect, $E_{Hill}$ is the resulting effect from the Hill equation and $C$ is the concentration.

For a system with multiphasic inhibition (e.g., points of inflection in the curves, connected agonist and antagonist effects), a simple Hill equation does not properly describe the complex binding behavior between enzyme and inhibitor. For this reason, an extended model is introduced, where each phase is considered as a part of a consecutive reaction to yield the total effect $E(C)$. It can be obtained as the product of the effect of every partial process $E_i(C)$.

$$E(C) = \Pi_i^n E_i(C) \tag{7}$$

$E_i(C)$ is written,

$$E_i(C; E_{\infty i}, EC_{50i}, H_i) = 1 + \frac{E_{\infty i} - 1}{1 + \left(\frac{EC_{50i}}{C}\right)^{H_i}} \tag{8}$$

The general equation is,

$$E(C; E_{\infty i} \dots E_{\infty n}, EC_{50i} \dots EC_{50n}, H_i \dots H_n) = \Pi_i^n \left(1 + \frac{E_{\infty i} - 1}{1 + \left(\frac{EC_{50i}}{C}\right)^{H_i}}\right) \tag{9}$$

Although there is no theoretical limit, in the software the case with $n = 3$ is applied, which proved to be suitable to explain most experimental data. It is (*E(C)** is the total effect, when *n* = 3):

$$E(C)^* = \left(1 + \frac{E_{\infty\,1} - 1}{1 + \left(\frac{EC_{50\,1}}{C}\right)^{H_1}}\right)\left(1 + \frac{E_{\infty\,2} - 1}{1 + \left(\frac{EC_{50\,2}}{C}\right)^{H_2}}\right)\left(1 + \frac{E_{\infty\,3} - 1}{1 + \left(\frac{EC_{50\,3}}{C}\right)^{H_3}}\right) \tag{10}$$

To obtain the relative effect as the final fitting equation, it has to be divided by $E_{max}$:

$$\frac{E(C)}{E_{max}} = \left(1 + \frac{E_{\infty\,1} - 1}{1 + \left(\frac{EC_{50\,1}}{C}\right)^{H_1}}\right)\left(1 + \frac{E_{\infty\,2} - 1}{1 + \left(\frac{EC_{50\,2}}{C}\right)^{H_2}}\right)\left(1 + \frac{E_{\infty\,3} - 1}{1 + \left(\frac{EC_{50\,3}}{C}\right)^{H_3}}\right) \tag{11}$$

An optimization algorithm was applied using this fit equation to optimize the parameters. For all POT inhibitors here, a monophasic dose-response curve was found.

# 7 Determination of $K_i$- and $\alpha$-parameter through Lineweaver-Burk plot

In a Lineweaver-Burk plot, the reciprocal reaction velocities $v$ are plotted against the reciprocal substrate concentration $[S]$, where for every inhibitor concentration a straight line is yielded[16]. As described by Breibeck *et al.*[17], the slopes $m$ of the Lineweaver-Burk lines and the intercepts $t$ with the ordinate can be used to calculate estimates for $K_i$ and the $\alpha$-value for subsequent non-linear regression, where

$$\frac{1}{v_{app}} = \frac{K_M(1+\frac{[I]}{K_i})}{v_{max}} * \frac{1}{[S]} + \frac{1}{v_{max}}\left(1+\frac{[I]}{\alpha K_i}\right) = m * \frac{1}{[S]} + t \qquad (12)$$

describes the double-reciprocal plot. For further evaluation of $m$ and $t$,

$$m = \frac{K_M}{v_{max}} * \frac{1}{K_i}[I] + \frac{K_M}{v_{max}} \qquad (13)$$

and

$$t = \frac{1}{v_{max}\,\alpha\,K_i}\,[I] + \frac{1}{v_{max}} \qquad (14)$$

were used. The intersection points with the ordinate in these equations yield values for $-K_i$ and $-\alpha \cdot K_i$, respectively, and allow for extraction of starting values of $K_i$ and the $\alpha$ for equation (5) in section 6.

In Lineweaver-Burk plots, the slopes of the lines and the location of the intersection points enable unambiguous determination of the inhibition type. All POTs in this study exhibited an intersection point of the three lines to the left of the y-axis and below the x-axis, indicating mixed-type inhibition and a value for $\alpha < 1$. The results imply that the inhibitors bind both to the free enzyme $E$ and to the enzyme-substrate complex $ES$, where the enzyme-substrate complex or subsequent species are preferred.

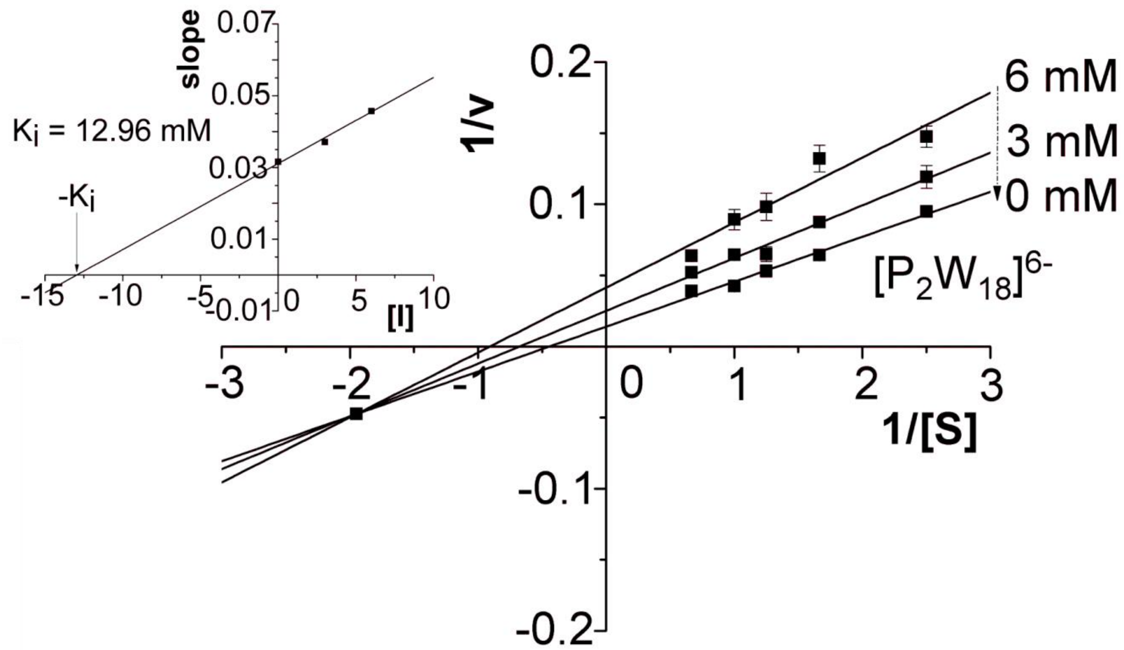


**Figure S14.** Kinetic evaluation of *Ab*PPO4 inhibition by **$[P_2W_{18}]^{6-}$** using a Lineweaver-Burk plot and evaluation of $K_i$-value *via* slopes of the curves plotted against inhibitor concentration (Table S4). The plot shows mixed-type inhibition, since the intersection point lies in the third quadrant. The inset illustrates the inhibitor concentration plotted against the slopes of the straight lines of the Lineweaver-Burk plot (Table S5).

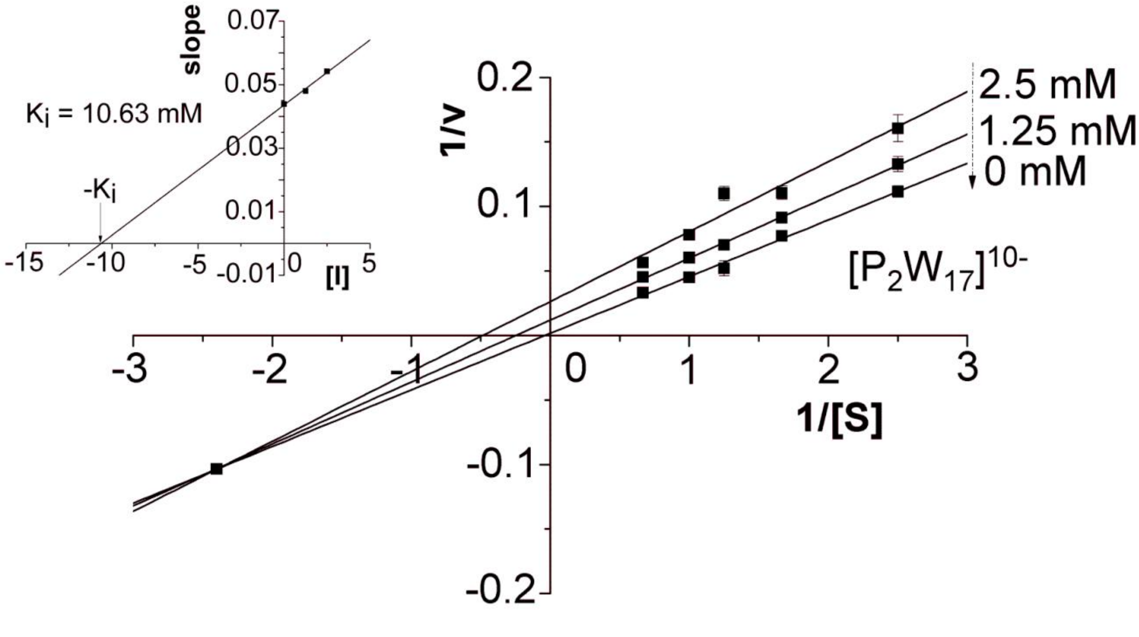


**Figure S15.** Kinetic evaluation of *Ab*PPO4 inhibition by **$[P_2W_{17}]^{10-}$** using a Lineweaver-Burk plot and evaluation of $K_i$-value via slopes of the curves plotted against inhibitor concentration (Table S4). The plot shows mixed-type inhibition, since the intersection point lies in the third quadrant. The inset illustrates the inhibitor concentration plotted against the slopes of the straight lines of the Lineweaver-Burk plot (Table S5).

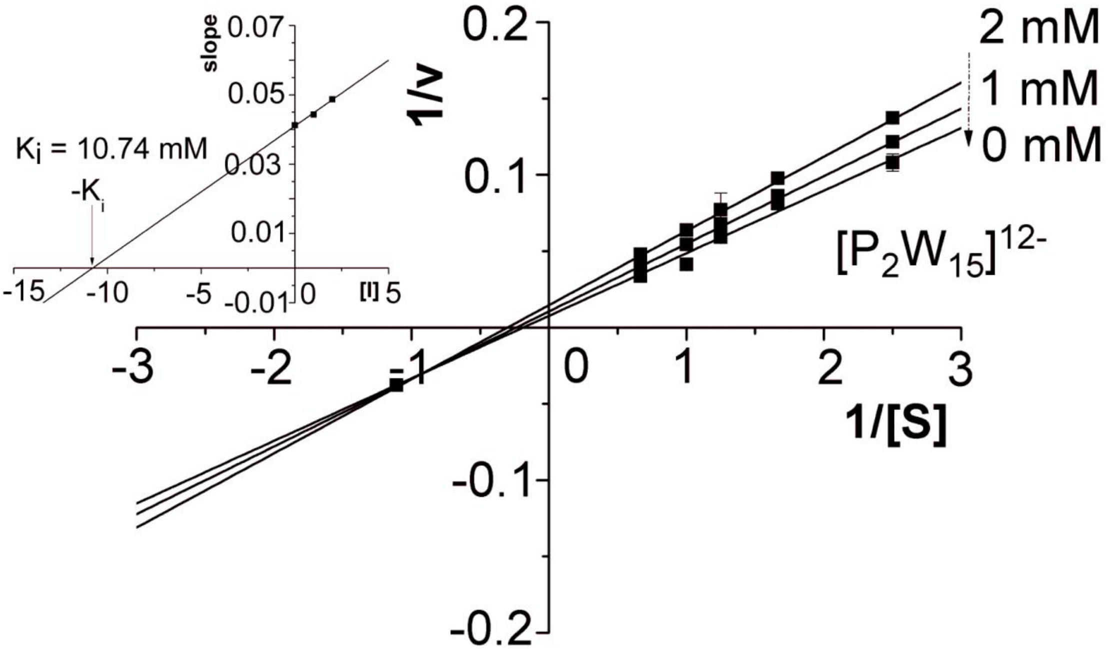


**Figure S16.** Kinetic evaluation of *Ab*PPO4 inhibition by **$[P_2W_{15}]^{12-}$** using a Lineweaver-Burk plot and evaluation of $K_i$-value via slopes of the curves plotted against inhibitor concentration (Table S4). The plot shows mixed-type inhibition, since the intersection point lies in the third quadrant. The inset illustrates the inhibitor concentration plotted against the slopes of the straight lines of the Lineweaver-Burk plot (Table S5).

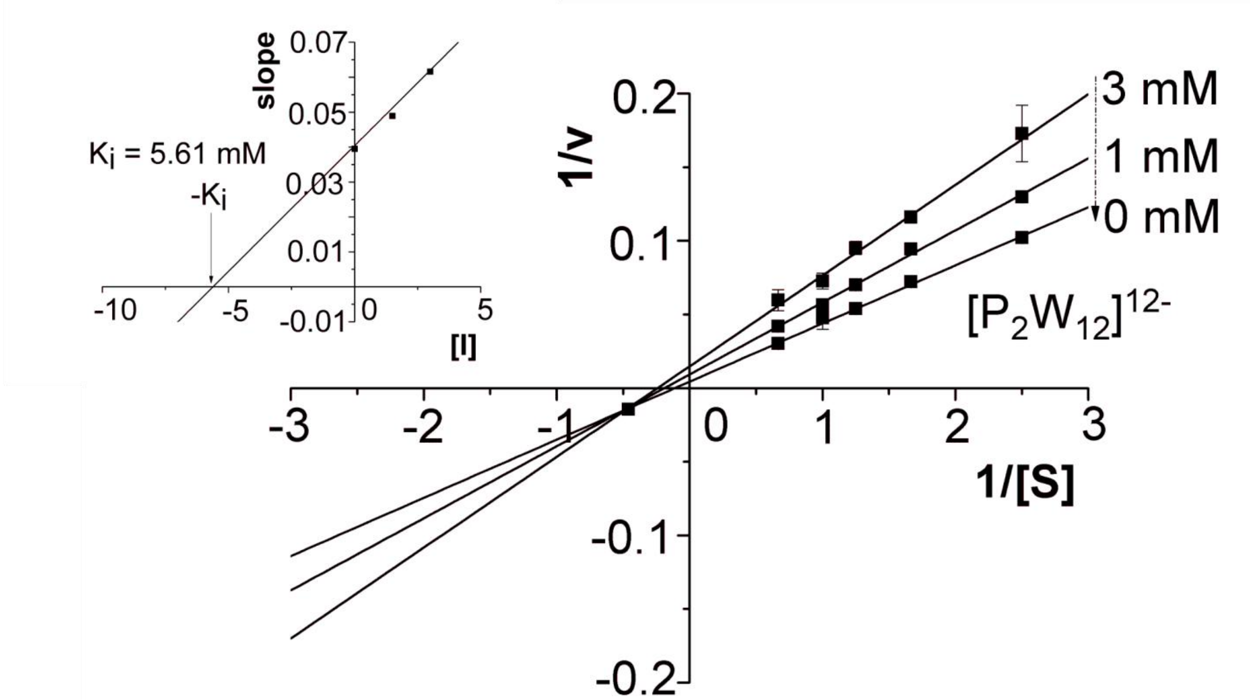


**Figure S17.** Kinetic evaluation of *Ab*PPO4 inhibition by **$[P_2W_{12}]^{12-}$** using a Lineweaver-Burk plot and evaluation of $K_i$-value *via* slopes of the curves plotted against inhibitor concentration (Table S4). The plot shows mixed-type inhibition, since the intersection point lies in the third quadrant. The inset illustrates the inhibitor concentration plotted against the slopes of the straight lines of the Lineweaver-Burk plot (Table S5).

**Table S4.** Kinetic evaluation of Lineweaver-Burk plots. m: slope, t: ordinate intersect, $R^2$: coefficient of determination (fraction of total variance of the dependent variable explained by the model), [I]: inhibitor concentration, [S]: substrate concentration.

| Inhibitor | Figure | [I] | [S] | m | t | $R^2$ |
|---|---|---|---|---|---|---|
| $[P_2W_{18}]^{6-}$ | S14 | 0 mM | 0.4-1.5 mM | 0.0316 | 0.014 | 0.98 |
| | | 3 mM | | 0.0371 | 0.0251 | 0.98 |
| | | 6 mM | | 0.0457 | 0.0415 | 0.92 |
| | Inset | 0-6 mM | | 0.0024 | 0.0311 | 0.98 |
| $[P_2W_{17}]^{10-}$ | S15 | 0 mM | 0.4-1.5 mM | 0.0439 | 0.0017 | 0.99 |
| | | 1.25 mM | | 0.048 | 0.0119 | 1.0 |
| | | 2.5 mM | | 0.0542 | 0.0263 | 0.95 |
| | Inset | 0-2.5 mM | | 0.0041 | 0.0436 | 0.99 |
| $[P_2W_{15}]^{12-}$ | S16 | 0 mM | 0.4-1.5 mM | 0.041 | 0.0077 | 0.99 |
| | | 1 mM | | 0.0443 | 0.0106 | 1.0 |
| | | 2 mM | | 0.0486 | 0.0147 | 0.99 |
| | Inset | 0-2 mM | | 0.0038 | 0.0408 | 0.99 |
| $[P_2W_{12}]^{12-}$ | S17 | 0 mM | 0.4-1.5 mM | 0.0395 | 0.0044 | 1.0 |
| | | 1.5 mM | | 0.0489 | 0.0093 | 1.0 |
| | | 3 mM | | 0.0616 | 0.0148 | 1.0 |
| | Inset | 0-3 mM | | 0.07202 | 0.0404 | 0.99 |

**Table S5.** Kinetic evaluation for $\alpha \cdot K_i$, m: slope, t: ordinate intersect, $R^2$: coefficient of determination, [I]: inhibitor concentration, [S]: substrate concentration.

| Inhibitor | $\alpha \cdot K_i$ | [I] | [S] | m | t | $R^2$ |
|---|---|---|---|---|---|---|
| $[P_2W_{18}]^{6-}$ | 2.85 | 0-6 mM | 0.4-1.5 mM | 0.0046 | 0.0131 | 0.99 |
| $[P_2W_{17}]^{10-}$ | 0.10 | 0-2.5 mM | | 0.0098 | 0.001 | 0.99 |
| $[P_2W_{15}]^{12-}$ | 2.14 | 0-2 mM | | 0.0035 | 0.0075 | 0.99 |
| $[P_2W_{12}]^{12-}$ | 1.58 | 0-3 mM | | 0.0034 | 0.005 | 0.98 |

## 8 UV-vis spectroscopic investigation

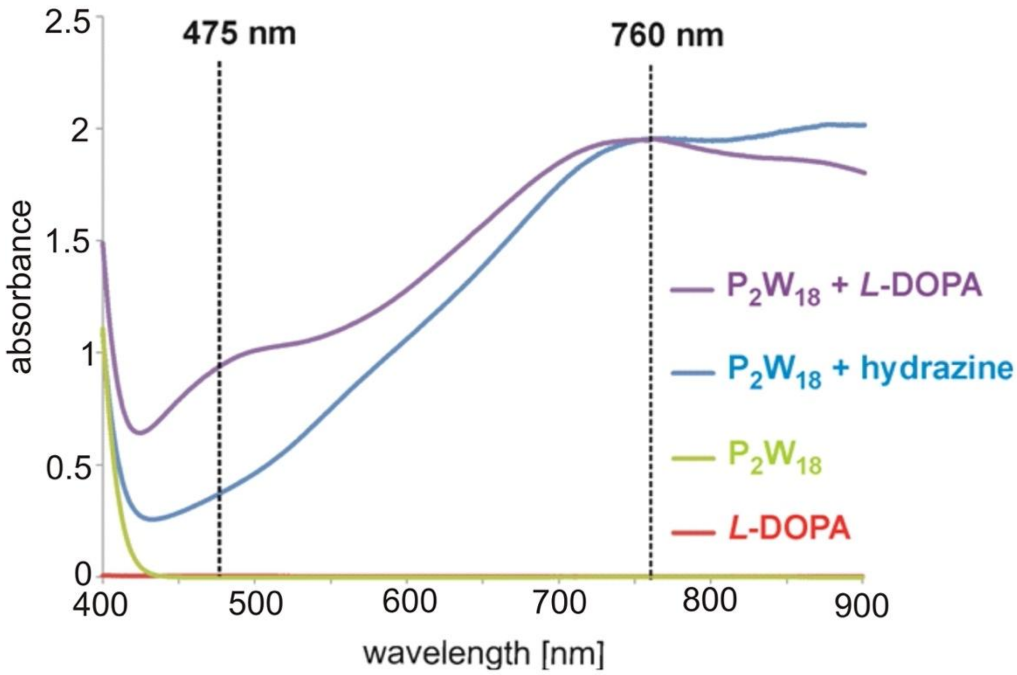


**Figure S18.** UV-vis spectra of 2 mM **$[P_2W_{18}]^{6-}$** (green), 2 mM **$[P_2W_{18}]^{6-}$** + 1 mM L-DOPA (purple), 2 mM **$[P_2W_{18}]^{6-}$** + excess of reducing agent hydrazine $N_2H_4{\cdot}2$ HCl (blue), 1 mM L-DOPA (red) recorded in 50 mM Na-citrate buffer at pH 6.8. The reduced POT species shows minimal absorbance at 475 nm, which was blanked out prior to the enzymatic activity measurements, and the additional rise in absorbance at 475 nm over the duration of 3 min was well assignable to dopachrome formation.

## 9 ESI-MS investigation

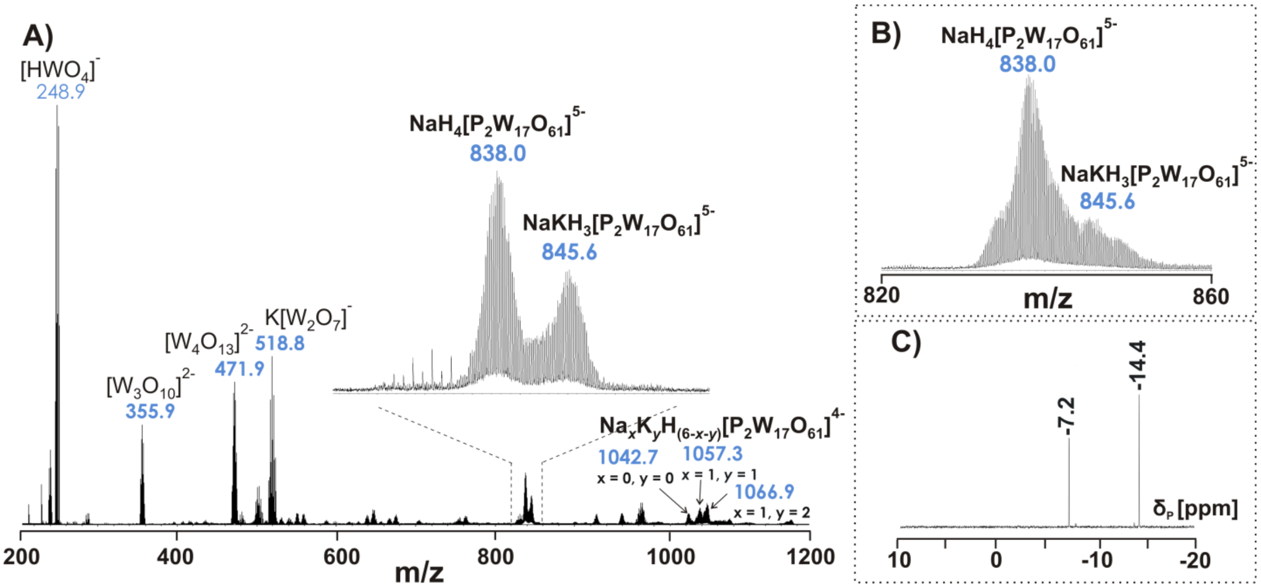


**Figure S19.** A) Negative ion-mode ESI-MS spectrum of $K_{10}[\alpha_2\text{-}P^{V}_2W^{VI}_{17}O_{61}]{\cdot}20H_2O$ in $H_2O$ (pH 6.4); B) fragment from the negative ion-mode ESI-MS spectrum of $K_{10}[\alpha_2\text{-}P^{V}_2W^{VI}_{17}O_{61}]{\cdot}20H_2O$ $CH_3CN/MeOH/H_2O$ (1 %) mixture; C) $^{31}P$-NMR spectrum of the solution used to measure mass spectrum A), showing the exclusive presence of $[\alpha_2\text{-}P^{V}_2W^{VI}_{17}O_{61}]^{10-}$ in solution.

## 10 Summary of POT charge densities of Keggin and Wells-Dawson POTs

In order to facilitate comparison of the inhibitory capacities of the Keggin and Wells-Dawson POT samples tested in our laboratory against *Ab*PPO4 and to obtain a structure-activity correlation, the charge densities (anionic charge per W addenda atom) of the species present before and after solubilization in physiological buffer were presented in Table S6. With regard to the charge densities, the following stability windows for POTs can be identified:

1) intact closed clusters: $q/m \approx 0.33 – 0.50$

Structures with higher charge densities were not analyzed. Structures with a *q/m* value lower than 0.33 are prone to hydrolysis or reduction under suitable conditions, both leading to an increase in charge density.

2) lacunary clusters: $q/m = 0.35 – 0.45$

Structures with lower charge densities were not analyzed. Structures with a *q/m* value higher than 0.45 achieve stabilization by taking up protons for charge compensation. However, too highly charged anions decompose and rearrange to more stable solution structures. Both of these pathways result in a decrease in charge density.

Overall, the preferred stable charge densities at neutral pH of both the intact and lacunary POTs investigated took values close to 0.4, and higher charge densities were only revealed as stable for intact clusters. This seems to be an intrinsic POT property as a combined result of 1) the reactivity of lacunary oxygen sites and 2) the hydrolytic susceptibility to hydroxide anions in aqueous solution in competition with electrostatic repulsion with the anionic POT charge. Notably, the size of the POT cluster does not play a role for these effects concerning single metal centers in their oxygen-bound environment.

The scenario is different when looking at the POT interaction with the enzyme *Ab*PPO4. Among the same-sized Keggin compounds exhibiting inhibitory effects on the enzyme, the apparent $K_i$ values show a clear correlation to the *q/m* values, with the lowest charge density corresponding to the highest protein affinity. But going to the larger Wells-Dawson archetype, comparing the active lacunary species $H_3[P^VW^{VI}_{11}O_{39}]^{4-}$ (Keggin) and $H_x[\alpha_2\text{-}P^V_2W^{VI}_{17}O_{61}]^{(10-x)-}$ ($x$ = 3 or 4) (Wells-Dawson), higher protein affinities were observed for the larger clusters despite of slightly higher charge densities. As stated in the main text, the basic inhibition effect shown by the Wells-Dawson clusters was revealed to correlate to the amount of $H_x[\alpha_2\text{-}P^V_2W^{VI}_{17}O_{61}]^{(10-x)-}$ ($x$ = 3 or 4) present in solution. The observed inhibition effect for the POT sample $[\mathbf{P_2W_{12}}]^{12-}$ seems to be caused by two lacunary species with suitable charge density.

**Table S6: Summary of Keggin and Wells-Dawson POT speciation from two studies.** The speciation of various Keggin POTs was taken from [17], the speciation of Wells-Dawson POTs is the result of the present study. Color code: orange, not stable; yellow, partially stable; light green, stabilized by protonation; green, stable; blue, obtained under long-term reducing conditions (3 days) in the presence of 1 mM *L*-DOPA. The POT species assigned to observed inhibitory capacity against *Ab*PPO4 are depicted in italics.

<table>
<tr><th>POT educt</th><th>Charge density q/m</th><th>Stability at pH 6.8</th><th colspan="2">Reaction to achieve stabilization</th><th colspan="2">POT product(s)</th><th colspan="2">Charge density q/m</th><th>K<sub>i</sub> [mM]</th></tr>
<tr><td>$[P^{V}W^{VI}_{12}O_{40}]^{3-}$</td><td>0.25</td><td>no</td><td colspan="2">hydrolysis</td><td colspan="2">$H_3[P^{V}W^{VI}_{11}O_{39}]^{4-}$</td><td colspan="2" rowspan="2">0.36</td><td>25.6</td></tr>
<tr><td>$[P^{V}W^{VI}_{11}O_{39}]^{7-}$</td><td>0.64</td><td>yes</td><td colspan="2">protonation</td><td colspan="2">$H_3[P^{V}W^{VI}_{11}O_{39}]^{4-}$</td><td>12.0</td></tr>
<tr><td>$[Si^{IV}W_{12}O_{40}]^{4-}$</td><td>0.33</td><td>partial</td><td>-</td><td>hydrolysis</td><td>$[Si^{IV}W^{VI}_{12}O_{40}]^{4-}$</td><td>$H_3[Si^{IV}W^{VI}_{11}O_{39}]^{5-}$</td><td>0.33</td><td>0.45</td><td>4.7</td></tr>
<tr><td>$[Si^{IV}W^{VI}_{11}O_{39}]^{8-}$</td><td>0.73</td><td>yes</td><td colspan="2">protonation</td><td colspan="2">$H_3[Si^{IV}W^{VI}_{11}O_{39}]^{5-}$</td><td colspan="2">0.45</td><td>-</td></tr>
<tr><td>$[B^{III}W^{VI}_{12}O_{40}]^{5-}$</td><td>0.42</td><td>yes</td><td colspan="2">-</td><td colspan="2">-</td><td colspan="2">-</td><td>-</td></tr>
<tr><td>$[Al^{III}W^{VI}_{12}O_{40}]^{5-}$</td><td>0.42</td><td>yes</td><td colspan="2">-</td><td colspan="2">-</td><td colspan="2">-</td><td>-</td></tr>
<tr><td>$[Al^{III}W^{VI}_{11}O_{39}]^{9-}$</td><td>0.82</td><td>no</td><td colspan="2">rearrangement</td><td colspan="2">$[Al^{III}W^{VI}_{11}O_{39}\{Al(H_2O)\}]^{6-}$</td><td colspan="2">0.50</td><td>54.1</td></tr>
<tr><td>$[H_2W^{VI}_{12}O_{40}]^{6-}$</td><td>0.50</td><td>yes</td><td colspan="2">-</td><td colspan="2">-</td><td colspan="2">-</td><td>-</td></tr>
<tr><td>$[Be^{II}W^{VI}_{12}O_{40}]^{6-}$</td><td>0.50</td><td>yes</td><td colspan="2">-</td><td colspan="2">-</td><td colspan="2">-</td><td>-</td></tr>
<tr><td>$[P^{V}_2W^{VI}_{18}O_{62}]^{6-}$</td><td>0.33</td><td>no</td><td>hydrolysis</td><td>reduction</td><td>$H_x[\alpha_2\text{-}P^{V}_2W^{VI}_{17}O_{61}]^{(10-x)-}$ (x = 3 – 4)</td><td>$H_2[P^{V}_2W^{VI}_{12}W^{V}_6O_{62}]^{10-}$</td><td>0.35 for x = 4 and 0.41 for x = 3</td><td>0.56</td><td>9.7</td></tr>
<tr><td>$[P^{V}_2W^{VI}_{17}O_{61}]^{10-}$</td><td>0.59</td><td>yes</td><td colspan="2">protonation</td><td colspan="2">$H_x[\alpha_2\text{-}P^{V}_2W^{VI}_{17}O_{61}]^{(10-x)-}$ (x = 3 – 4)</td><td colspan="2">0.35 for x = 4 and 0.41 for x = 3</td><td>6.5</td></tr>
<tr><td>$[P^{V}_2W^{VI}_{15}O_{56}]^{12-}$</td><td>0.80</td><td>no</td><td colspan="2">rearrangement</td><td colspan="2">$H_x[\alpha_2\text{-}P^{V}_2W^{VI}_{17}O_{61}]^{(10-x)-}$ (x = 3 – 4)</td><td colspan="2">0.35 for x = 4 and 0.41 for x = 3</td><td>13.6</td></tr>
<tr><td>$H_2[P^{V}_2W^{VI}_{12}O_{48}]^{12-}$</td><td>1.00</td><td>no</td><td colspan="2">rearrangement</td><td colspan="2">$[H_2P^{V}_2W^{VI}_{12}O_{48}]^{12-}$ + $H_x[\alpha_2\text{-}P^{V}_2W^{VI}_{17}O_{61}]^{(10-x)-}$ + $[P^{V}_2W^{VI}_{19}O_{69}(H_2O)]^{14-}$ + $[P^{V}_8W^{VI}_{48}O_{184}]^{40-}$</td><td colspan="2">/</td><td>7.5</td></tr>
</table>

## 11 Two isomers of *α*-[$P_2W_{17}$]$^{10-}$

The pH-adjusted sample [$P_2W_{12}$]$^{12-}$ is reported to initially rearrange towards the $\alpha_1$-lacunary isomer[5] (Figure S20), and then more slowly to the thermodynamically stable $\alpha_2$-isomer[18].

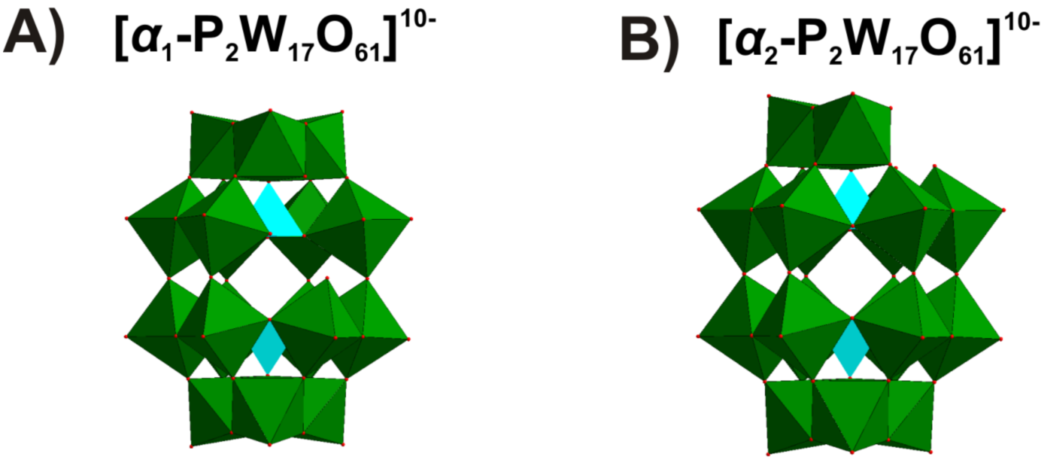


**Figure S20.** Two *α*-isomers A) & B) of [$P_2W_{17}$]$^{10-}$ are given. [$\alpha_1$-$P_2W_{17}$]$^{10-}$ has a missing equatorial {WO} group ("belt") whereas in [$\alpha_2$-$P_2W_{17}$]$^{10-}$ the void is in polar position ("crown")[4]. Above pH 5 in aqueous solution, isomerization of [$\alpha_1$-$P_2W_{17}$]$^{10-}$ to [$\alpha_2$-$P_2W_{17}$]$^{10-}$ occurs.4 *Color code:* green: {$WO_6$} octahedra; turquoise: {$PO_4$} tetrahedron.

## 12 Abbreviations

**Table S7.** Abbreviations used in the supplementary information.

| | |
|---|---|
| Å | Ångström |
| *Ab* | *Agaricus bisporus* |
| ACN | Acetonitril |
| Da | Dalton |
| FA | Formic acid |
| FWHM | Full width at half maximum |
| HPLC | High pressure liquid chromatography |
| *L*-DOPA | *L*-3,4-dihydroxyphenylalanine |
| mM | Millimolar |
| NMR | Nuclear magnetic resonance |
| POT | Polyoxotungstate |
| PPO | Polyphenol oxidase |
| TFA | Trifluoroacetic acid |

**Table S8**. Abbreviations used for the Wells-Dawson POTs tested in this study.

| Solid compound | Anion | POT in Na-citrate buffer at pH 6.8 | POT in Na-citrate buffer at pH 6.8 in the presence of *L*-DOPA |
|---|---|---|---|
| $Cat_6[\alpha/\beta\text{-}P^{V}{}_2W^{VI}{}_{18}O_{62}]\cdot 14H_2O$ (Cat = $K^+$, $NH_4^+$) | $[\alpha/\beta\text{-}P^{V}{}_2W^{VI}{}_{18}O_{62}]^{6-}$ | $[\mathbf{P_2W_{18}}]^{6-}$ | $[\mathbf{P_2W_{18}}]^{6-}$ + *L*-DOPA |
| $K_{10}[\alpha_2\text{-}P^{V}{}_2W^{VI}{}_{17}O_{61}]\cdot 20H_2O$ | $[\alpha_2\text{-}P^{V}{}_2W^{VI}{}_{17}O_{61}]^{10-}$ | $[\mathbf{P_2W_{17}}]^{10-}$ | $[\mathbf{P_2W_{17}}]^{10-}$ + *L*-DOPA |
| $K_{12}[P^{V}{}_2W^{VI}{}_{15}O_{56}]\cdot 24H_2O$ | $[P^{V}{}_2W^{VI}{}_{15}O_{56}]^{12-}$ | $[\mathbf{P_2W_{15}}]^{12-}$ | $[\mathbf{P_2W_{15}}]^{12-}$ + *L*-DOPA |
| $(NH_4)_{12}[H_2P^{V}{}_2W^{VI}{}_{12}O_{48}]\cdot 24H_2O$ | $[H_2P^{V}{}_2W^{VI}{}_{12}O_{48}]^{12-}$ | $[\mathbf{P_2W_{12}}]^{12-}$ | $[\mathbf{P_2W_{12}}]^{12-}$ + *L*-DOPA |

## 13 References


1. Pretzler, M., Bijelic, A. & Rompel, A. Heterologous expression and characterization of functional mushroom tyrosinase (*Ab*PPO4). *Sci. Rep.* **7**, srep1810, https://doi.org/10.1038/s41598-017-01813-1 (2017).

2. Lima, C. R. *et al.* Combined kinetic studies and computational analysis on kojic acid analogs as tyrosinase inhibitors. *Molecules* **19**, 9591–9605, https://doi.org/10.3390/molecules19079591 (2014).

3 Saenger, W. *Handbook of Proteolytic Enzymes*, Rawlings N. D. & Salvesen, G. Academic Press, New York, 3240–3242 (2013).

4. Pope, M. T. Heteropoly and Isopoly Oxometalates; Springer: Berlin, Germany, 1983.

5. Ginsberg, A. P. *Inorganic synthesis*. **27**, 104-111, (1990).

6. Dawson, B. The Structure of the 9(18)-Heteropoly Anion in Potassium 9(18)-Tungstophosphate, $K_6[P_2W_{18}O_{62}]14H_2O$. *Acta Cryst.* **6**, 113, https://doi.org 10.1107/S0365110X53000466 (1952).

7. Chen, Y.-G., Gong, J. & Qu, L.-Y. Tungsten-183 nuclear magnetic resonance spectroscopy in the study of polyoxometalates. *Coord. Chem. Rev.* **248**, 245–260, https://doi.org/10.1016/j.cct.2003.11.003 (2004).

8. Müller, A. *et al.* „Adding“ stable functional complementary, nucleophilic and electrophilic clusters: a synthetic route to $[\{(SiW_{11}O_{39})\}]Mo_3S_4(H_2O)_3(\mu\text{-}OH)\}_2]^{10-}$ and $[\{(P_2W_{17}O_{61})Mo_3S_4(H_2O)_3(\mu\text{-}OH)\}_2]^{14-}$ as examples. *Chem. Commun.* **13**, 1189-1190 https://doi.org/10.1039/a903170g (1999).

9. Finke, R. G., Lyon, D. K., Nomiya, K. & Weakley, T. J. R. Structure of nonasodium – triniobatopentadecawolframatodiphosphate-acetonitrile-water (1/2/23), $Na_9[P_2W_{15}Nb_3O_{62}]$ $2CH_3CN$ $23H_2O$. *Acta Cryst.* **C46**, 1592-1596, https://doi.org/10.1107/S0108270190000038 (1990).

10. Mal, S. S. & Kortz, U. The wheel-shaped $Cu_{20}$ tungstophosphate $[Cu_{20}Cl(OH)_{24}(H_2O)_{12}(P_8W_{48}O_{184})]^{25-}$ ion. *Angew. Chem. Int. Ed.* **24**, 3777-3780, https://doi.org/10.1002/anie.200500682 (2005).
11. Boyd, T., Mitchell, S. G., Gabb, D., Long, D.-L. & Cronin, L. Investigating cation binding in the polyoxometalate-super-crown $[P_8W_{48}O_{184}]^{40-}$. *Chem. Eur. J.* **17**, 12010-12014, https://doi.org/10.1002/chem.201101666 (2011).
12. Maksimovskaya, R. I. & Maksimov, G. M. $^{31}P$ NMR studies of hydrolytic conversions of 12-tungstophosphoric heteropolyacid. *Coord. Chem. Rev.* **385**, 81-99, https://doi.org/10.1016/j.ccr.2019.01.014 (2019).
13. Haouas, M. *et al*. Investigation of the protonation state of the macrocyclic $\{H_nP_8W_{48}O_{184}\}$ anion by modeling $^{183}W$ NMR chemical shifts. *New J. Chem.* **41**, 6112-6119, https://doi.org/ 10.1039/c7nj00915a (2017).
14. Copeland, R. A. Evaluation of enzyme inhibitors in drug discovery. A guide for medicinal chemists and pharmacologists. *Methods Biochem. Anal.* **46**, 1-265, https://doi.org/10.1002/9781118540398 (2013).
15. Di Veroli, G. *et al.* An automated fitting procedure and software for dose-response curves with multiphasic features. *Sci. Rep.* **5**, 14701, https://doi.org/10.1038/srep14701 (2015).
16. Lineweaver, H. & Burk, D. The determination of enzyme dissociation constants. *J. Am. Chem. Soc.* **56**, 658-66, https://doi.org/10.1021/ja01318a036 (1934).
17. Breibeck, J., Gumerova, N. I., Boesen, B. B., Galanski, M. & Rompel, A. Keggin-type polyoxotungstates as mushroom tyrosinase inhibitors - A speciation study. *Sci. Rep.* **9**, 5183, https://doi.org/10.1038/s41598-019-41261-7 (2019).
18. Bartis, J. *et al.* Lanthanide complexes of the $\alpha_1$-isomer of the $[P_2W_{17}O_{61}]^{10-}$ heteropolytungstate: Preparation, stoichiometry, and structural characterization by $^{183}W$ and $^{31}P$ NMR spectroscopy and europium(III) luminescence spectroscopy. *Inorg. Chem.* **38**, 1042-1053, https://doi.org/10.1021/ic980384i (1999).
